# Exploring Protein Conformational Changes Using a Large‐Scale Biophysical Sampling Augmented Deep Learning Strategy

**DOI:** 10.1002/advs.202400884

**Published:** 2024-10-10

**Authors:** Yao Hu, Hao Yang, Mingwei Li, Zhicheng Zhong, Yongqi Zhou, Fang Bai, Qian Wang

**Affiliations:** ^1^ Department of Physics University of Science and Technology of China Hefei Anhui 230026 China; ^2^ Shanghai Institute for Advanced Immunochemical Studies and School of Life Science and Technology ShanghaiTech University 393 Middle Huaxia Road Shanghai 201210 China; ^3^ School of Information Science and Technology ShanghaiTech University 393 Middle Huaxia Road Shanghai 201210 China; ^4^ Shanghai Clinical Research and Trial Center Shanghai 201210 China

**Keywords:** conformational changes, deep learning, proteins, transition pathway

## Abstract

Inspired by the success of deep learning in predicting static protein structures, researchers are now actively exploring other deep learning algorithms aimed at predicting the conformational changes of proteins. Currently, a major challenge in the development of such models lies in the limited training data characterizing different conformational transitions. To address this issue, molecular dynamics simulations is combined with enhanced sampling methods to create a large‐scale database. To this end, the study simulates the conformational changes of 2635 proteins featuring two known stable states, and collects the structural information along each transition pathway. Utilizing this database, a general deep learning model capable of predicting the transition pathway for a given protein is developed. The model exhibits general robustness across proteins with varying sequence lengths (ranging from 44 to 704 amino acids) and accommodates different types of conformational changes. Great agreement is shown between predictions and experimental data in several systems and successfully apply this model to identify a novel allosteric regulation in an important biological system, the human β‐cardiac myosin. These results demonstrate the effectiveness of the model in revealing the nature of protein conformational changes.

## Graphical abstract

A large‐scale database of protein conformational changes is constructed using molecular dynamics simulations. Building on this dataset, the present study develops a deep learning model that can predict the transition pathways of various proteins, including those with simple opening/closing transitions as well as those exhibiting significant global changes in folding topology.

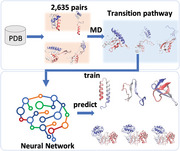

## 1 Introduction

The structure of a protein determines how it interacts with the external environment and ultimately determines its function in various biological processes.^[^ ^1^ ^]^ Consequently, the identification of protein structures, whether through experimental^[^ ^2^ ^]^ or theoretical methods,^[^ ^3^ ^]^ has attracted considerable attention in recent decades. Since experimental methods can be time and resource consuming, there is a great need for theoretical algorithms to predict protein structures. Notably, the recent groundbreaking advancements made by AlphaFold2^[^ ^4^ ^]^ have instilled confidence in the potential of deep learning techniques to attain this objective. However, AlphaFold2 primarily focuses on predicting the static native structure of a protein rather than its conformational changes. While the native state is undeniably important, many proteins must undergo partial or global conformational changes to fulfill their functions.^[^ ^5^ ^]^ To date, accurately predicting the transition pathway of proteins remains a major challenge.

One important way for probing protein conformational changes is the elastic network‐based normal mode analysis,^[^ ^6^ ^]^ noted for its simplicity and robustness across many systems. Additionally, advanced hybrid methods that combine elastic network models and short molecular dynamics simulations^[^ ^7^ ^]^ have shown improved performance. However, further evaluation is necessary to assess the applicability of these models to proteins undergoing complex conformational changes, such as fold‐switching. Another different approach is to develop deep learning models, inspired by AlphaFold2. However, a significant bottleneck in the development of such models is the severe lack of training data. On one hand, current experimental techniques face difficulties in capturing the transient structures along the transition pathway. On the other hand, simulating large protein conformational changes by conventional molecular dynamics simulations is also challenging due to high free energy barriers in the transition state. In contrast to static structure prediction, which can draw on more than 200 000 high quality structures in the Protein Data Bank,^[^ ^8^ ^]^ there are currently few large‐scale databases available for structural information along the transition pathway.

A popular solution to address the data shortage is the latent space embedding.^[^ ^9^ ^]^ For a protein with two distinct experimentally resolved structures, people utilize various deep learning frameworks, such as auto‐encoders,^[^ ^10^ ^]^ Boltzmann generators,^[^ ^11^ ^]^ or diffusion models,^[^ ^12^ ^]^ to project the structural ensembles near the two structures into two regions (*A_l_* and *B_l_*) in a low dimensional latent space. The embedding is then accompanied by a key assumption that the transition pathway follows a linear interpolation between *A_l_* and *B_l_*. While these studies have provided valuable insights and demonstrated promising results in specific systems, the general theoretical basis remains unclear. Why can we always assume that the pathway must be linear in the latent space? In this work, we will show that in certain cases, this assumption may require reconsideration.

We adopted a more direct approach to address the data shortage. By combining molecular dynamics simulations^[^ ^13^ ^]^ with enhanced sampling method,^[^ ^14^ ^]^ we extensively simulated the conformational change of 2635 proteins, and directly obtained structures that compose the transition pathway. Leveraging this dataset, we trained a general deep learning model to predict the structural information along the transition pathway of a given protein. To prove the effectiveness of the model, we will first compare the predictions with experimental or all‐atomistic simulation data across multiple well‐established systems. Subsequently, we will apply the model to explore an important biological system that has not yet been thoroughly understood, leading to the identification of a new allosteric regulation. We believe that the model can improve our understanding of protein conformational changes.

## 2 Results

### 2.1 Creating Single‐State (SS) and Multi‐State (MS) Protein Datasets

**Figure 1:**
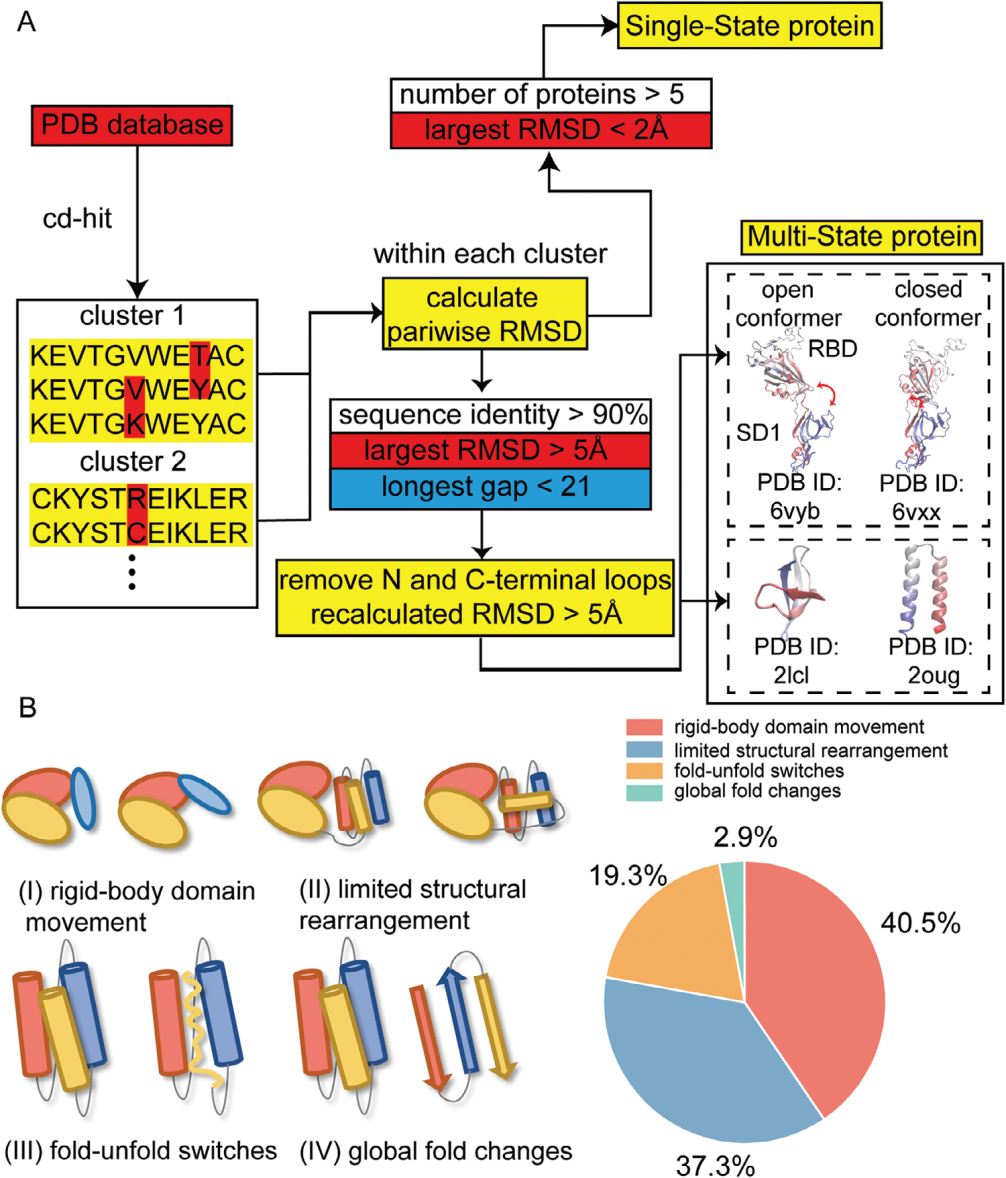
Creation of the Single‐ (SS) and Multi‐State (MS) protein datasets. A) Workflow of the dataset creation. B) Statistic analysis of the four categories in the MS dataset.

The creation of the SS and MS protein datasets involves a comprehensive search and comparison of the structures in the Protein Data Bank (PDB), as depicted in **Figure** **1**A.

The SS dataset, comprising a total of 3454 entries, includes proteins that have highly converging conformations observed in experimental studies (detailed information can be found in Experimental Section). In contrast, the MS dataset comprises 2635 proteins (each protein has two structures in PDB) that show significant conformational changes, defined as a root‐mean‐square deviation (RMSD) of >5 Å. This dataset can be further categorized into four subgroups^[^ ^15^ ^]^ (Figure 1B).

In Category I, the conformational changes mainly result from the relative movement between different domains, while the structure of each individual domain remains largely unchanged. An example is the SARS‐CoV‐2 spike protein,^[^ ^5d^ ^]^ which can switch between “open” (PDB ID: 6vyb) and “closed” (PDB ID: 6vxx) due to the relative movement between the receptor binding domain (RBD) and the S1 domain. However, neither the RBD nor the S1 domain itself undergoes significant structural alterations. In Category II, conformational changes originate from the relative movement of distinct segments within the same domain. In Category III, the protein experiences a localized unfolding transition, with certain region shifting from a helical structure to a coil, or from a beta‐sheet structure to a coil. In Category IV, the protein undergoes a global alteration in its folding topology, often accompanied by a secondary structure switch between helix and beta sheet. An example is the protein RfaH,^[^ ^5b^ ^]^ in which the C‐terminal segment can change from all‐helical state (PDB ID: 2oug) to all‐beta state (PDB ID: 2lcl). Of these four subgroups, the first two comprise the majority of the MS dataset at 40.5% and 37.3%. The third and fourth subgroups, which are referred to as “fold‐switching proteins”^[^ ^16^ ^]^ or “metamorphic proteins”^[^ ^17^ ^]^ in the literature, make up 22.2% of the MS dataset.

To identify the residues that are important for protein conformational changes, we performed two statistic studies. First, we calculated the abundance of each type of residue contact in the MS dataset and compared them with those in the SS dataset. The results showed that certain types of residue contacts, such as ARG‐GLU, GLN‐GLU, and GLN‐GLN (Table S1, Supporting Information), which contain relatively long and flexible side chains, are more abundant in the MS proteins. Second, for the MS dataset, we calculated the frequency of each contact type when these contacts are present in one state but broken in the other. Again, residues with long flexible side chains were found to occur more frequently (Table S2, Supporting Information). These consistent results indicate that residues with long and flexible side chains play a pivotal role in protein conformational changes. This is likely due to their ability to engage in the formation/breakage of specific interactions, e.g., ionic locks or hydrogen bonds between the side chains (or backbone), which may be related to movements of domains or secondary structural elements.

### 2.2 Calculating the Free Energy Landscape and the Transition Pathway of MS Proteins

**Figure 2:**
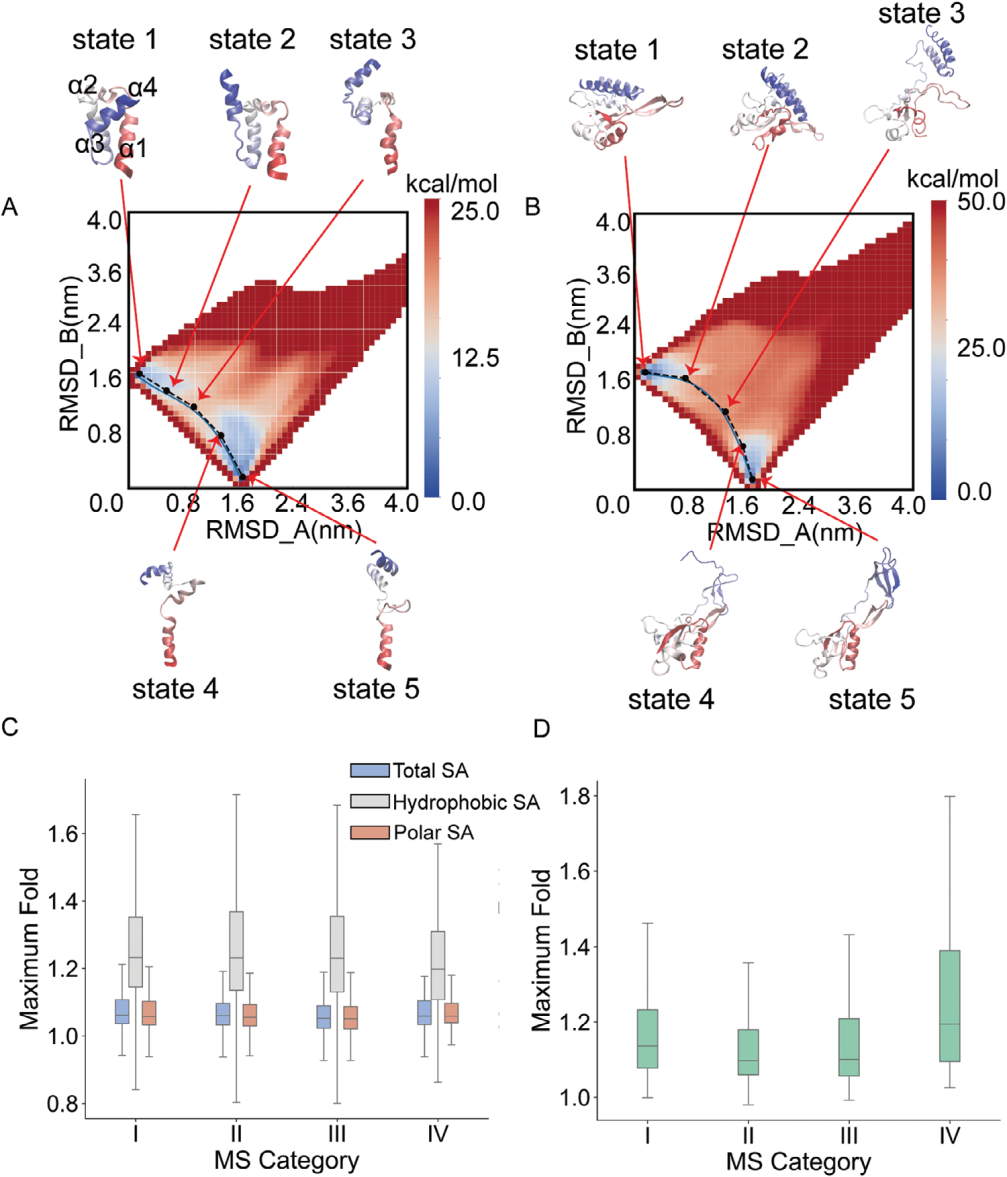
Investigation of the transition pathway of proteins with multi‐conformations. A,B) 2D free energy landscape and the transition pathway for (A) the protein Transcription Enhancer Factor 1(TEF‐1) and (B) the protein RfaH. The collective variables are RMSD_A and RMSD_B, representing the root‐mean‐square deviation of simulated structures with respect to experimentally resolved structure A (TEF‐1: PDB ID: 2hzd, residue 11 to 71; RfaH: PDB ID: 2oug, residue 3 to 155) and structure B (TEF‐1: PDB ID: 4z8e; RfaH: PDB ID: 6c6s), respectively. Only Cα atoms are used in the calculations. C) Box plots showing the maximum fold changes in the solvent accessibility (SA) across four MS protein categories (I–IV). The types of SA are represented as Total SA (blue), Hydrophobic SA (gray), and Polar SA (salmon). D) Box plots showing the maximum fold changes in the radius of gyration (*R_g_*, green) of proteins across four transition categories.

We employed a combination of structure‐based coarse‐grained models and metadynamics simulations to comprehensively analyze the 2D free energy landscapes of all 2635 proteins in the MS datasets. Subsequently, we searched within these landscapes to identity the transition pathway connecting two distinct conformational states of each MS protein (Please see Methods). Despite its simplicity, the structure‐based model has consistently demonstrated its effectiveness in studying conformational changes of proteins over the past decades.^[^ ^18^ ^]^ Here, we present two additional illustrative examples. The first example is transcription enhancer factor 1 (TEF‐1), which represents a conformational change characterized by a straightforward open‐closed transition (**Figure** **2**A). TEF‐1 consists of four alpha helices, with α3 and α4 (colored in blue) able to both bind to and detach from α1 (colored in red). Our simulation captures the gradual separation of α3 and α4 from α1 (state 1 → state 2 → … → state 5).

The second example is the previously mentioned protein RfaH. This case poses a considerably greater challenge because the C‐terminus of RfaH undergoes a classical folding transition from helix to beta. As depicted in Figure 2B, the two distinct minima of free energy (state 1 and state 5) accurately represent the two structures resolved in experiments (the position of RMSD_A = 0 or RMSD_B = 0). More importantly, the simulation results reveal that the transition begins with a gradual detachment of the C‐terminus from the remaining domains (state 1 → state 2 → state 3), proceeds through the unfolding in the C‐terminus (state 4), and ultimately ends in the transformation from helix to beta (state 5). The transition state of the entire pathway is state 3, signifying that the detachment of the C‐terminus serves as the central trigger for this transition. This observation is consistent with previous experimental findings.^[^ ^19^ ^]^ More examples and comparisons with experimental data can be found in the following sections.

To elucidate the underlying patterns of conformational changes in MS proteins, we conducted a statistical analysis of all transition pathways identified through simulations. Coarse‐grained structures were initially reconstructed into all‐atom structures, followed by energy minimization to ensure the structural validity. Assessment using MolProbity^[^ ^20^ ^]^ revealed that the recovered structural quality is good (Figure S1, Supporting Information). Subsequently, we calculated the maximum total, polar, and hydrophobic surface areas (SA) and the radius of gyration (*R*_g_) for various MS proteins using these optimized structures.

Figure 2C,D illustrates the maximum total, polar, hydrophobic surface area (SA), and the *R*_g_ of four types of MS proteins in the pathways compared to the experimentally resolved structures. Most proteins exhibit an increasing trend in these characteristics. Notably, the distribution of the maximum hydrophobic shows greater changes compared to the total and polar SA, indicating that the buried hydrophobic cores tend to become exposed and thus serve as a more sensitive indicator of protein conformational changes (Figure 2C). Additionally, similar patterns of SA changes are observed across four transition categories. However, Category IV proteins show the most significant *R*_g_ changes (Figure 2D), suggesting that these proteins undergo a more extended state during transitions due to a large change in the folding topology. In contrast, Category II and III, which correspond to intra‐domain structural rearrangements and local unfolding/folding, respectively, exhibit smaller *R*_g_ changes.

### 2.3 Predicting the High Energy State of MS Proteins

Our ultimate goal is to construct a deep learning model capable of predicting the structural information along the transition pathway for MS proteins. To achieve this, we will initially develop a core module that focuses on the key point within the pathway, then extend its capabilities. A natural choice for the key point is the transition state. Therefore, we used the transition state data from simulations to train a neural network capable of predicting such state for a given protein. We note that, due to the use of a coarse‐grained model, the transition state obtained in simulations, while providing valuable information during conformational changes (as illustrated in Figure 2B), may not precisely match the real transition state in the strict definition, but rather represent a high free energy state nearby. To avoid confusion, we refer to this module as the High Energy State Predictor (HESpre).

**Figure 3:**
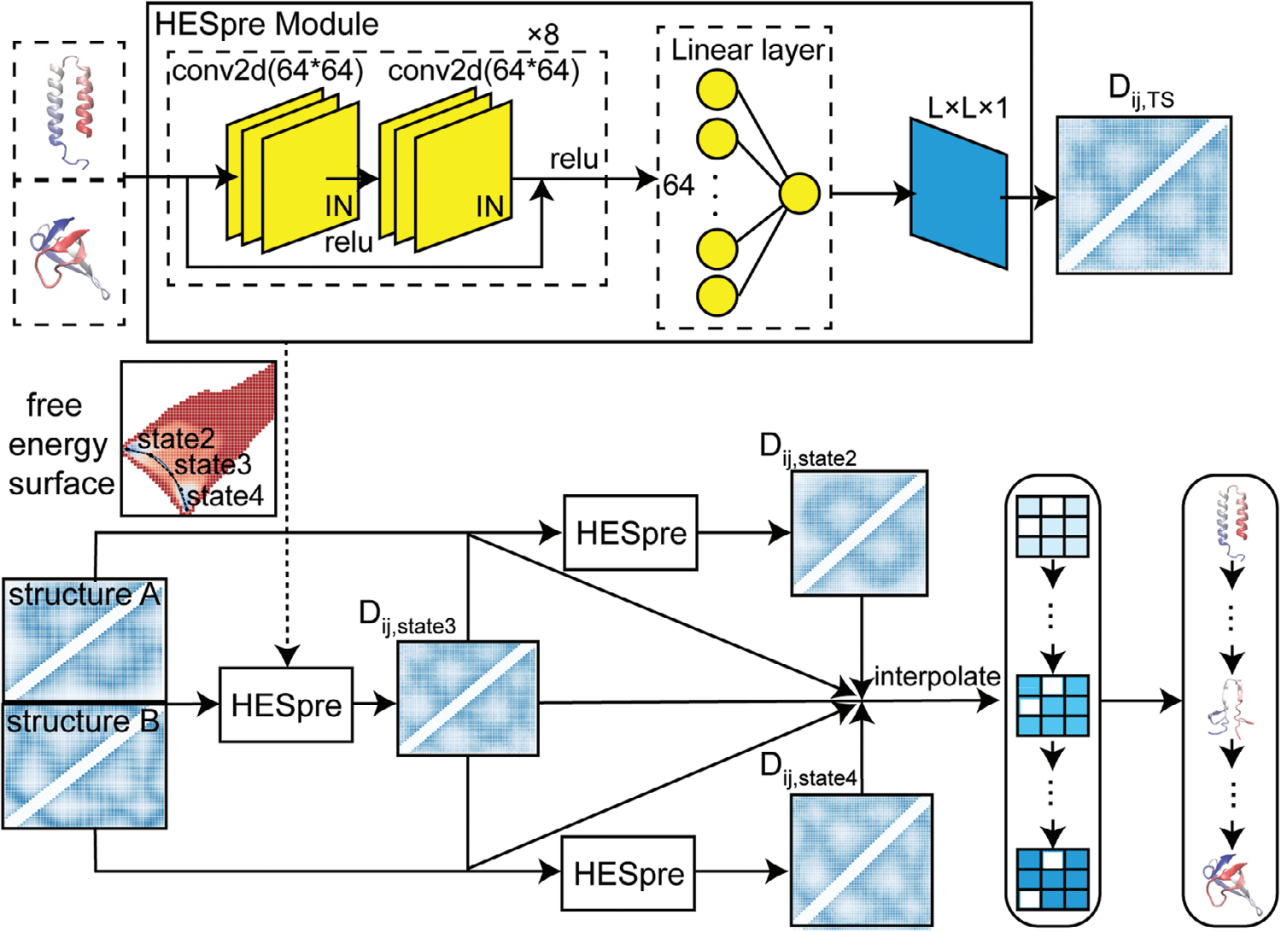
Neural network architecture to predict the transition pathway of proteins with multi‐conformations. The core module is named HESpre, with the objective of predicting the structural information in the high energy state. This module is then used iteratively to predict the entire pathway. The full model is named PATHpre. *D_ij_* represents the pairwise residue distance matrix.

For any MS protein, HESpre Module (**Figure** **3**) utilizes the structural information of its two conformational states to predict the pairwise distance matrix *D*_*ij*,*TS*_ for the high energy state in the transition pathway (please see Equation (5) and other details in Methods). *D_ij_* ≥ 0.5 signifies the formation of a contact between residue *i* and *j* (defined as the averaged distance between two residues less than 10Å). *D*_*ij*,*TS*_ decreases as the probability of the contact breaking increases. There are two kinds of contact formations in a MS protein: those that exist in both states (common) and those that only exist in one state (unique). When assessing the model performance, we only consider *D*_*ij*,*TS*_ for the unique contacts, as the common contacts barely change in the entire transition pathway. For unique contacts, there is a very low correlation (*r* = 0.168) between the Euclidean distance matrix and the sequence distance matrix, as shown in Figure S2 (Supporting Information).

The reliability of HESpre is confirmed by a strong Pearson correlation between its predictions on *D*_*ij*,*TS*_ and the actual simulation data (*r* = 0.77), along with a low mean absolute error (MAE = 0.044) in the testing dataset (**Table** **1**). To assess the model's applicability across different scenarios, we further partitioned the testing dataset based on three distinct criteria: the sequence length, the structural characteristics (fold‐switching or no fold‐switching), and the RMSD between two distinct conformational states. The model exhibits similar performance in all subgroups, underscoring its versatility and robustness. The scattering plots were provided in Figure S3 (Supporting Information). In addition, we have selected 8 proteins as illustrative examples to highlight the consistency between our predictions and the simulation results at the molecular level (Figure S4, Supporting Information).

**Table 1:** Comparison between our predictions on the distance matrix *D*_*ij*,*TS*_ and the simulation data in different scenarios. The Pearson correlation (*r*) and the mean absolute error (MAE) are represented.

	All	N_length <200	N_length 200–400	N_length >400	Fold switching	No fold switching	RMSD >1 nm	RMSD <1 nm
*r*	0.77	0.83	0.75	0.69	0.81	0.76	0.80	0.69
MAE	0.044	0.049	0.044	0.039	0.065	0.042	0.051	0.039

### 2.4 Predicting the Transition Pathway of MS Proteins

The concept of HESpre module can be applied iteratively, making it straightforward to predict the distance matrix *D_ij_* for the entire pathway (Figure 3). In short, for an MS protein with two distinct experimentally resolved structures A and B, we can leverage the information of A and B to obtain *D_ij_* for the high energy state 3, then utilize the information of A and state 3 to calculate *D_ij_* for the intermediate state 2, and similarly, employ the information of state 3 and B to determine *D_ij_* for the intermediate state 4. Therefore, we retrained the neural network to simultaneously fit *D_ij_* for states 2, 3, and 4. Our predictions exhibit a strong correlation with the actual simulation data in the testing dataset (*r* = 0.80 for state 2; *r* = 0.71 for state 3; *r* = 0.78 for state 4). The scattering plots were provided in Figure S5 (Supporting Information). *D_ij_* for other intermediate points along the transition pathway can be determined through interpolation. In the last step of the model, we convert each *D_ij_* to a 3D structure by performing short simulations with adding *D_ij_* as distance restraints (Please see details in Experimental Section). This model is labeled as PATHpre.

**Figure 4:**
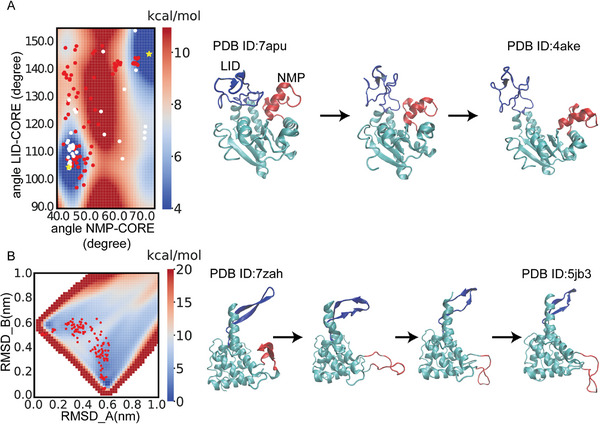
Applying PATHpre to study the conformational changes of two proteins: A) adenylate kinase and B) 30S ribosomal protein S7. Projections of our generated conformers onto the free energy landscape are represented by red dots. The free energy landscape profiles are from metadynamics simulations. In panel (A), multiple experimentally resolved structures are represented by white dots. Two specific structures (PDB ID: 7apu and 4ake) used as the input of our model are represented by yellow asterisks. The angle NMP‐Core is defined as the angle formed by the geometric centers of three protein segments: residues 115–125, 90–100, and 35–55. The angle LID‐Core is defined as the angle formed by the geometric centers of three protein segments: residues 179–185, 115–125, and 125–153.

We first evaluated PATHpre in a well‐established system, adenylate kinase (AdK). We projected our generated conformations onto the free energy landscape spanned by the angle movements of the LID and NMP domains (**Figure** **4**A, red dots). It indicates that the transition of AdK from the closed state to the open state involves unsynchronized movements of the two domains, starting with the opening of the LID, followed by the opening of the NMP. This result is consistent with the free energy landscape from previous all‐atomistic simulations,^[^ ^21^ ^]^ which suggests that the free energy barrier for opening the NMP is higher than that for opening the LID. Additionally, there is consistency between our predicted conformers (red dots in Figure 4A) and several crystal structures of AdK (white dots in Figure 4A). Applications to two other examples, HIV‐1 protease and Phosphoglycerate kinase (PGK), can be found in Figure S6 (Supporting Information). Following previous studies,^[^ ^22^ ^]^ we projected our generated conformers onto the subspace spanned by experimental ePC1 and ePC2 (Figure S6, Supporting Information). It can be observed that these generated conformers cover the experimental structures well in both the open and closed ensembles, as well as the transition between them.

Noticeably, a hybrid methods based on the elastic network model (ENM) and short molecular dynamics simulations, ClustENMD,^[^ ^7c^ ^]^ is also highly effective in describing protein conformational changes between the open and closed states.^[^ ^22^ ^]^ To demonstrate the advantage of our model, we compared two methods in another system, 30S ribosomal protein S7. The conformational change of this protein involves two regions: the twist of one beta sheet (Figure 4B, colored in blue), as well as local unfolding and reorientation of another beta sheet (Figure 4B, colored in red). Our method successfully captures the transitions in both regions (Figure 4B). In contrast, ClustENMD did not capture the unfolding process of the red beta sheet well (Figure S7A, Supporting Information). If ClustENMD initiates from one structure (PDB ID:7zah), this region will maintain the beta sheet‐like conformation; while if the algorithm initiates from the other structure (PDB ID: 5jb3), this region will maintain the coil conformation. However, we emphasize that ClustENMD is an unbiased technique where the intermediate states are not forced toward a target structure. Therefore, searching the pathway is inherently more challenging. For proteins with more complex conformational changes such as fold‐switching, our method offers a greater advantage. We will discuss this in the next section.

### 2.5 In Comparison with Experimental Data or All‐Atomistic Simulation Results

In this section, we present three distinct scenarios in which our model can enhance our understanding of conformational changes in proteins, accompanied by a comparison with experimental data or all‐atomistic simulations.

**Figure 5:**
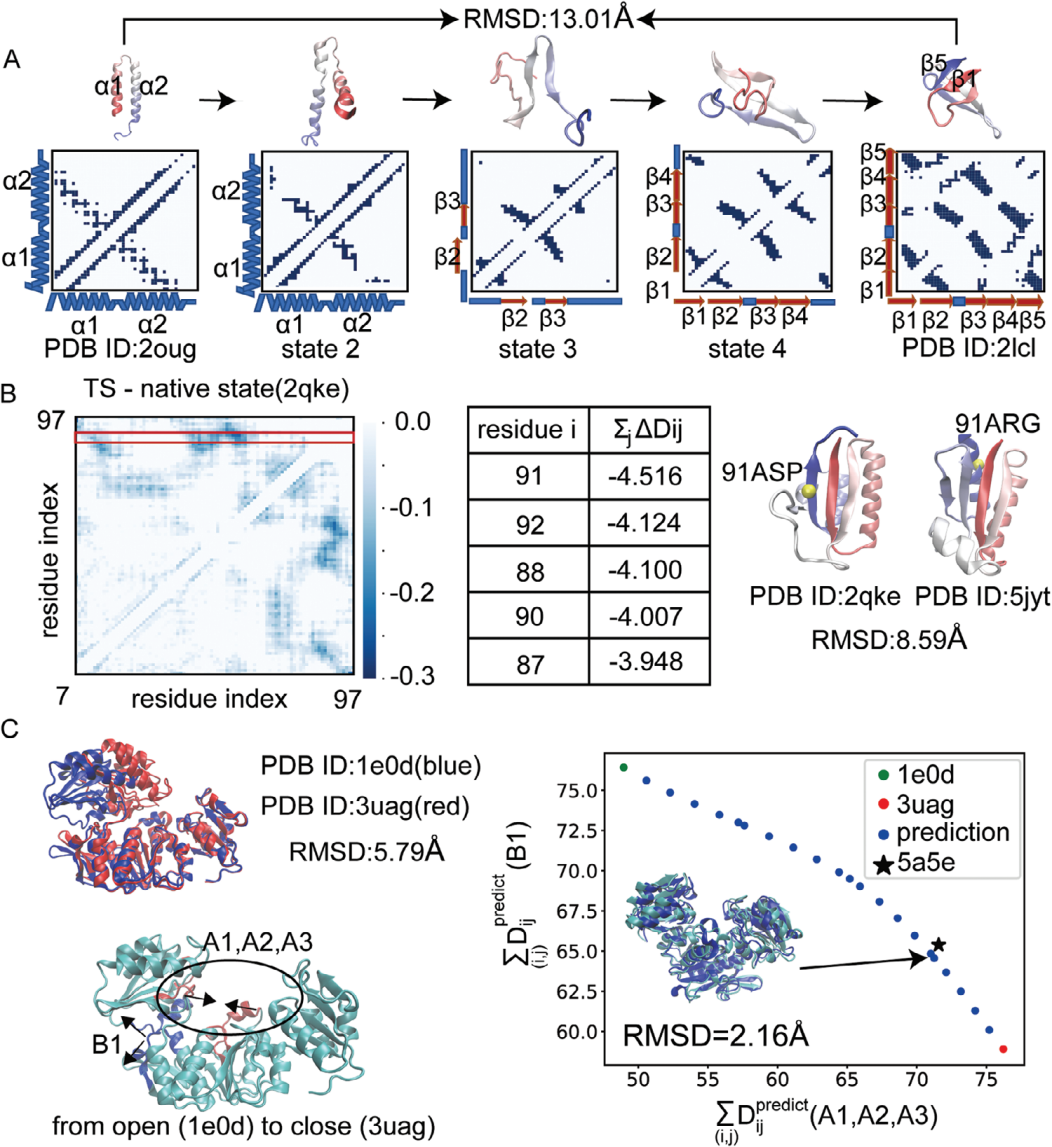
Comparing model predictions with experimental data or all‐atomistic simulation results. A) Folding pathway of the C‐terminus of RfaH. B) Difference distance matrix Δ*D*_*ij*,*TS* − *native*_ between the high‐energy state and the native state for the protein KaiB. Table shows the most important residues influencing the conformational change in predictions. C) Predicted transition pathway of protein MurD from the open to the closed state. A1 represents all possible contact formations between two segments: residues 111–115 and residues 316–325. A2: contacts between residues 176–185 and residues 320–325. A3: contacts between residues 181–188 and residues 346–352. B1: contacts between residues 256–270 and residues 324–335. In the right panel, X‐axis represents summarizing all *D_ij_* in the A1, A2 and A3 regions. Y‐axis represents summarizing all *D_ij_* in the B1 region. Blue dots represent our predicted transition pathway. Black star represents the crystal structure of the intermediate state (PDB ID: 5a5e). Left corner shows the alignment of the predicted structure (cyan) and this crystal structure (blue). RMSD shows the root‐mean‐square deviation between the two, calculated with Cα beads.

- Determining the order of conformational changes. There are a large number of conformational changes involving both unfolding and re‐folding processes. To illustrate, consider the case of the C‐terminus of RfaH (**Figure** **5**A), which undergoes a conformational change involving the unfolding of two helices and the re‐folding of five beta sheets. An important question is in which order these beta‐sheets are folded. Due to the free energy barrier in the conformational change, this type of system presents a big challenge for conventional molecular dynamics simulations. In contrast, our model efficiently generates contact maps sequentially along the transition pathway within seconds and takes another 15 minutes for reverse‐mapping from coarse‐grained structures to high quality atomistic ones (the latter depends on the size of the protein). It indicates that the transition should commence with contact breaking between α1 and α2 (state 2), followed by the formation of the β2‐β3 sheet (state 3), and subsequently, the formation of the β2‐β3‐β4 sheets (state 4), ultimately culminating in the formation of the β1‐β5 sheet (state 5). This order aligns with multiple previous all‐atomistic simulations,^[^ ^23^ ^]^ which also suggested that the β1‐β5 sheet should be the final step in this transition. NMR studies also showed that β1‐β5 has the least hydrogen bonding formations during this conformational change.^[^ ^19^ ^]^ We also applied two ENM‐based hybrid methods, ClustENMD^[^ ^7c^ ^]^ and coMD^[^ ^7b^ ^]^ on this system. However, the conformers generated by ClustENMD are concentrated around the two initial structures without significant deformations (Figure S7B, Supporting Information), while coMD generated an unrealistic bent helical structure (Figure S7C, Supporting Information). Furthermore, after 500 cycles of coMD, while the contacts between β3 and β4 of RfaH were broken, the contacts between β1 and β5 remained intact (Figure S7C, Supporting Information). This is inconsistent with the re‐folding order observed in previous studies, as discussed above.
- Identifying important residues regulating the transition. The rationale behind this application lies in the assumption that if the contacts associated with a particular residue are greatly lost in the transition state compared to the native state, this residue is likely to play a pivotal role in the transition. To illustrate, in the case of protein KaiB, we employed HESpre to derive the difference distance matrix Δ*D*_*ij*,*TS* − *native*_ between the high‐energy state and the native state (Figure 5B, only counting negative values). Subsequently, for each residue *i*, we summarized Δ*D*_*ij*,*TS* − *native*_ for all *j* (Figure 5B, red square) as a metric to indicate the significance of residue *i* in the conformational transition. The results show that residue 91 has the greatest influence on the transition. This prediction is consistent with previous experimental results showing that mutation at residue 91 accelerates the transition rate by a factor of 4–5.^[^ ^24^ ^]^
- Determining the 3D intermediate structure. Consider the protein MurD,^[^ ^25^ ^]^ which can shift from open (PDB ID: 1e0d) to closed (PDB ID: 3uag). Experimental data has also identified the crystal structure of its intermediate state (PDB ID: 5a5e).^[^ ^26^ ^]^ During the transition from open to closed, there are three regions where contacts significantly increase (Figure 5C, A1–A3) and one region where contacts are lost (Figure 5C, B1). To visualize the transition pathway in a 2D space, we summarize all *D_ij_* in the A1, A2 and A3 domains as one reaction coordinate and summarize all *D_ij_* in the B1 domain as the other. We employed PATHpre to predict *D_ij_* along the transition pathway and projected it into these two coordinates (Figure 5C, blue dots). Remarkably, the experimentally identified intermediate state (PDB ID: 5a5e, black star) aligns with our predicted pathway very well. In addition, our structural prediction resembles the experimental data (RMSD = 2.16Å, Figure 5C).

One important characteristics of our model is to be able to label the transition state of protein conformational changes, thus providing useful information to understand the thermodynamics of this process. For protein RfaH and MurD, after generating structural ensembles for three intermediate states along the transition pathway by our deep learning model, we projected those structures back to the free energy landscape and labeled the state we predicted as the transition state (Figure S8, Supporting Information). For both proteins, these intermediates are energetically separable. In addition, for RfaH, the transition state happens after the denaturation of two helices but before the refolding of beta sheets, indicating that the helices denaturation is the key for this transition. For MurD, the conformational change involves a relatively straightforward open‐closed motion. The free energy increases as contacts break in region B1. Following the formation of new contacts in regions A1–A3, the free energy begins to decrease.

#### 2.5.1 Application: Identifying Allosteric Regulations in Human β‐Cardiac Myosin

**Figure 6:**
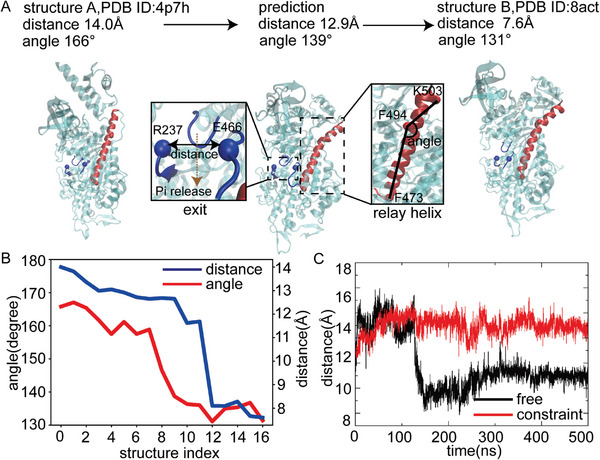
Model application in human β‐cardiac Myosin. A) Distinct structures resolved by experiments and the intermediate states predicted by our model PATHpre. The size of the exit is measured by the Cα‐Cα distance between R237 and E466, as two residues form strong electrostatic interactions in a “closed” exit. The bending of the relay helix is measured by the angle among K503, F494, and F473. B) Changes in distance and angle along the predicted transition pathway. PATHpre generated 15 structures sequentially along the transition pathway from one state to the other. For convenience, we labeled these structures from 1 to 15, with index 0 and 16 representing two experimentally resolved structures. C) Validation from all‐atomistic simulations. Black curve represents that the relay helix is free to bend. Red curve represents that the relay helix is constraint in a straight conformation. Another repeated simulation was provided in Figure S9 (Supporting Information).

In this section, we aim to show that the application of our model in a biologically important system, human β‐cardiac Myosin, helps to identify a new allosteric regulation associated with heart disease. Myosin consumes ATP to generate tensions for heart contraction^[^ ^27^ ^]^ and an abnormal ATPase rate resulting from genetic mutations can lead to a severe heart disease known as hypertrophic cardiomyopathy.^[^ ^28^ ^]^ Consequently, finding a way to regulate the ATPase rate offers new insights for the treatment of the disease. It has been reported that the rate‐limiting step of ATP turnover is the phosphate release,^[^ ^29^ ^]^ a process controlled by the size of the exit (**Figure** **6**A, colored in blue). Therefore, we aim to discover a mechanism that can regulate this size.

A simple comparison of the two conformational states (Figure 6A, structure A and structure B) of myosin reveals that associated with the size reduction in the exit, another distal domain named the relay helix (Figure 6A, colored in red) becomes bent. However, static structures alone cannot tell the order of these two events. Here, we fill in the missing information by applying PATHpre to predict the transition pathway from structure A (larger exit) to structure B (smaller exit). Our results have captured an important intermediate state where the relay helix is bent but the exit remains large (Figure 6A, the middle panel). Further analysis on the predicted states sequentially distributed along the transition pathway clearly reveals that the bending of the relay helix occurs before the exit reduction (Figure 6B). These findings consistently support that the change in the relay helix might serve as a prerequisite for the size reduction in the exit, implying an allosteric regulation mechanism. To test this hypothesis, we performed all‐atomistic simulation and found that the size of the exit no longer decreases when the relay helix is kept in a straight conformation (Figure 6C, red versus black), which validates the allosteric regulation role of the relay helix.

## 3 Discussion

In this study, we have simulated the large conformational change (RMSD > 5Å) of 2635 proteins. It is noticeable that in certain cases the RMSD is not sufficient to distinguish intermediate metastable conformations. In Figure S10 (Supporting Information), we provided some examples that require specifically designed collective variables to distinguish these conformations. These data have been integrated into our database. However, given our goal of establishing a large‐scale database for protein conformational changes, designing personalized collective variables for each protein is impractical. Additionally, as we showed in Figure S11 (Supporting Information), RMSD is still adequate for distinguishing metastable conformations in certain cases. Thus, RMSD serves as an efficient general collective variable for our purpose. With all simulations, we generated a database with structural information along the transition pathway for each protein. This database, as well as the deep learning model built upon this database, although originating from a smooth energy landscape, still offers important structural information consistent with experimental data or all‐atom simulations in multiple testing systems (Figure 2B and Figures 4, 5, 6). These systems encompass not only proteins that undergo simple opening/closing transitions, but also proteins with substantial global changes in folding topology. Consequently, we believe that the database can serve as a valuable approximation or constraint in the development of predictive models for protein conformational changes to alleviate the current problem of data insufficiency.

It is interesting to investigate whether the transition pathway in the configuration space is linear in the latent space, as assumed in many studies.^[^ ^10^, ^11^, ^12^ ^]^ We found that this assumption should be used with caution. In Figure S12A (Supporting Information), we project the actual transition pathway of protein AdK identified in simulations (black curve) onto a 2D latent space, following previous research.^[^ ^10a^ ^]^ It turns out that the transition pathway is non‐linear (black versus orange). This is because the conformational change of this protein involves the open‐closed movements of two isolated domains (LID and NMP). These movements are not synchronized, introducing non‐linearity into the system. The order of movement from multiple regions is challenging to capture for a deep learning model that only receives structural information near the native state. Another example is fold‐switching proteins which completely changes the folding topologies (Figure S12B, Supporting Information). Simulation shows that the contacts between β1 and β5 should form in the last stage of conformational change,^[^ ^23^ ^]^ which cannot be reproduced with a linear interpolation in the latent space (Figure S12B, Supporting Information, red line).

Building on this database, we developed a general deep learning model that leverages two distinct structures of a random protein to predict the transition pathway. The applications of this model encompass, but are not limited to, predicting the order of conformational changes (Figure 5A), investigating the key residues regulating the transition (Figure 5B), determining the intermediate 3D structure (Figure 5C), and identifying potential allosteric regulations (Figure 6). Due to the inherent nature of coarse‐grained representations, the accuracy of the model decreases as the conformational changes become subtle. This is also reflected in the enhanced model performance for proteins with an RMSD greater than 1 nm during transitions, compared to those with an RMSD less than 1 nm (Table 1). Consequently, this study primarily focuses on proteins with RMSD greater than 5 Å during transitions. However, we randomly selected 70 proteins with RMSD between 2 and 5 Å in transitions and compared our predictions with simulation results. The resulting correlation coefficient is 0.69 (Figure S13, Supporting Information). When comparing ENM‐based (physics‐based) methods, we observe that these methods are effective for proteins with open‐closed transitions, but our model demonstrates advantages in handling proteins with more complexed conformational changes (It is important to note that our comparison did not cover all existing ENM‐based methods, as many are not open access). Besides, our model has two additional benefits. First, the use of deep‐learning techniques enables efficient prediction. Although we perform reconstruction to all‐atomistic structures, each point along the transition pathway can be reconstructed independently, thus allowing parallel processing. Second, our prediction labels the high free energy states near the transition, which can be helpful in identifying critical residues, as illustrated in Figure 5B.

Recently, several other deep learning models related to protein conformational changes have emerged. Our model has the following characteristics. First, our model is trained with transition data derived from simulations, eliminating the assumption that the transition pathway is linear in the latent space. Second, our model is a generalized one that applies to all proteins, eliminating the necessity for custom retraining for each individual protein.^[^ ^10^ ^]^ Third, our model focuses on large conformational changes (RMSD > 5Å) for proteins with well‐defined structures, rather than intrinsically disordered proteins^[^ ^30^ ^]^ or proteins with relatively small local changes which are important in drug design.^[^ ^31^ ^]^ The database and the source code will be updated to https://github.com/qwang897/PATHpre.

## 4 Experimental Section

### Data Collection

All protein structures were downloaded in the Protein Data Bank (PDB) before October 2022. The sequence length was required to be >50. First, CD‐hit^[^ ^32^ ^]^ was utilized to perform a clustering analysis for these proteins based on sequence similarity. The cutoff was set to 80%. Then, within each cluster, if two members simultaneously satisfy three conditions: A) sequence similarity >90%; B) the root‐mean‐standard‐deviation (RMSD) >5Å (N‐ and C‐terminal loops are excluded from RMSD calculations); C) the longest gap <21 residues, they are considered as two distinct conformations of the same protein and added to the Multi‐State (MS) protein dataset. If three or more members satisfy those conditions, the two with the highest sequence similarity are chosen as representatives. On contrary, if one cluster has more than five members, and the largest pairwise RMSD is <2 Å, all members are considered as Single‐State (SS) proteins, and the one with the shortest sequence is added to the SS dataset as a representative.

### Multi‐State (MS) Protein Classification

Based on previous research,^[^ ^15^ ^]^ MS proteins were categorized into four types: rigid‐body domain movement, limited structural rearrangement, fold‐unfold switches, and global fold changes. Category I is characterized by the relative motion between two domains while internal structures remain unchanged. Category II involves conformational changes due to relative movements within the same domain. Category III involves local unfolding transformations, where regions transition from helix or strand to loop structures. Category IV involves comprehensive alterations in folding topology, usually with transitions between helices and strands.

To differentiate between Category I and II, each protein was visually inspected to identify relative movements between domains (Category I) or within a domain (Category II). Comparing RMSD and *R_g_* between two representative structures of these two categories revealed that Category I MS proteins exhibited greater structural changes (Figure S14, Supporting Information). Next, DSSP program^[^ ^33^ ^]^ was used to assign the types of the secondary structure (helix, strand or loop) for each residue for the two representative states. Proteins with more than five residues changing from helix or strand to loop structures were classified as Category III. Meanwhile, proteins with more than five residues alternating between helix and β‐sheet structures were classified as Category IV.

### Minimization Protocol

All structures along transition pathway were optimized using the Prime module in Schrödinger 2024‐1, employing the S‐OPLS^[^ ^34^ ^]^ force field. The minimization procedure was set to “Automatic”, using conjugate gradient minimization for large gradients and changing to the truncated Newton method when gradients were sufficiently small. The RMSD gradient convergence threshold was set at 0.01 kcal/mol/Å. A maximum of 2 minimization cycles was allowed, with each cycle comprising up to 65 steps.

### Simulation Protocol

A coarse‐grained Cα model was used to simulate the conformational change of multi‐conformational proteins. For each protein in the MS dataset, all missing loops of its two distinct structures A and B were first filled in by the SWISS‐MODEL server,^[^ ^35^ ^]^ then used SMOG2.0^[^ ^36^ ^]^ to create a dual basin structure‐based Hamiltonian.^[^ ^37^ ^]^ The shadow contact map^[^ ^38^ ^]^ was used. The strength of the contact potential was set to 1.0 for all contacts without customized parameterization for each protein (more details can be found in Supporting Information). The 2D free energy landscape was calculated by well‐tempered metadynamics^[^ ^14^, ^39^ ^]^ along two reaction coordinates: the RMSD respective to structure A and that to structure B. A Gaussian was deposited every 500 timesteps, with the initial height equal to 1.0 kJ mol^−1^. The Gaussian width was set to 0.05. The bias factor was set to 50. All simulations were performed by an in‐house version of Gromacs^[^ ^40^ ^]^ with PLUMED.^[^ ^41^ ^]^ The Langevin equation was used to run simulations in a low friction limit. Each simulation last at least 4  ×  10^8^ steps. Two tests were performed to ensure the convergence of the metadynamics simulations. First, the free energy landscape was calculated with different simulation lengths (Figure S15A–C,E–G, Supporting Information) and observed minimal changes after 3  ×  10^8^ integration steps. Second, the free energy landscape was calculated twice for the same protein, initiating the simulation from its two distinct experimentally resolved structures, respectively. The results were quite similar (Figure S15C,D,G,H, Supporting Information). Representative time evolution profiles of collective variables were also shown in Figure S16 (Supporting Information). It can be seen that the system is able to diffuse in the collective variables (CVs) space. During the full‐length simulations (4  ×  10^8^ steps), by average there are 60 transitions sampled from state A to state B, or vice versa. These findings support that the metadynamics simulations have converged well. Converged landscapes tend to result in more consistent locations for the transition states.

All‐atomistic molecular dynamics simulations were used to study the allostery of human β‐cardiac Myosin. The initial structure was obtained from Protein Data Bank (PDB ID: 4p7h) and characterized by the AMBER ff14SB force filed^[^ ^42^ ^]^ in simulations. The systems were solvated in a cubic box with TIP3P water molecules, maintaining a box size of 15.5 Å. A salt concentration of 150 mm was employed. Van der Waals interactions were truncated at 10 Å and the electrostatic interactions were calculated using the PME method.^[^ ^43^ ^]^ Temperature was held at 300 K using the Langevin Thermostat.^[^ ^44^ ^]^ Two control systems were constructed: the first system is unbiased, while the other imposed constraints on the relay helix of myosin in a straight conformation. For each system, a 500‐ns simulation was performed using Amber‐2022^[^ ^45^ ^]^ and repeated once. Constraints were applied on the relay helix (residues 472–505) with a force constant of 200 kcal mol^−1^ Å^−2^ to maintain its straight conformation.

### Transition Pathway Identification

A two‐round search was performed to identify the transition pathway connecting two distinct structures A and B of the same protein in the free energy landscape. In the first round, the nudged elastic band (NEB) method^[^ ^46^ ^]^ was used. 7 beads were randomly distributed in the landscape. Adjacent beads were connected by a spring. After 1000 iterations with NEB, these beads were distributed along the pathway. This process was repeated 100 times. To ensure that the selection of the spring constant does not impact the identification of pathways, four distinct spring constants were adopted: 0.3, 0.6, 0.9, and 1.2 N nm^−^^2^, respectively. So, in this step there were a total of 400 possible paths. The second round followed an earlier work^[^ ^47^ ^]^ based on the local free energy difference. The landscape was divided into grids. The search started from the structure A and continued moving to one of the four adjacent grids with the probability $e^{-\Delta G/k_{B}T}$ until reaching the structure B. Here Δ*G* is the free energy difference between grids. This process was repeated 50 times. In this two‐round search, a total of 450 possible paths were found. The path with the lowest free energy barrier was identified as the transition pathway and its peak was determined as the transition state.

### Transition Pathway Analysis

Pulchra^[^ ^48^ ^]^ was used to covert coarse‐grained structures to all‐atom ones. To measure the degree of conformational change in an MS protein relative to the experimentally resolved structures, the study introduced a metric called "max fold” computed as follows:

(1) $$Max\;fold\;=\frac{\max\left(X_{path}\right)}{\max(X_{A},X_{B})}\;$$

*X* represents the total, polar, hydrophobic solvent accessible surface areas (SA) calculated by the module named Calculate Properties panel in Maestro (Schrödinger, Inc.), or the radius of gyration (*R*_g_) calculated by the script of calc_radgyr.py (Schrödinger, Inc.) *X_A_* (*X_B_*) represents *X* in the two distinct structures solved experimentally. *X_path_* represents *X* in the transition pathway identified by simulations. The probability files (Figure 2) were fitted by using the normal distribution.

The frequency of residue‐residue occurrence in the MS‐ or SS‐ dataset was determined as:

(2) $$\;F_{pq}=\frac{1}{n}\;\sum_{i\;=\;1}^{n}\frac{N_{pq}^{i}}{N_{p}^{i}N_{q}^{i}}$$

where *n* is the number of MS‐ or SS‐ proteins, *pq* is the contact type consisting of residue *p* and *q* (eg., Ala‐Ala, totally 210 types), $N_{pq}^{i}$ represents the overall count for all residue‐residue pair *pq*, with their separation ranging from 3 Å to 10 Å in protein *i*.$\;N_{p}^{i}$ and $N_{q}^{i}$ represents the count of residue *p* and *q* in protein *i*, respectively. The frequency ratio of each contact type in MS‐proteins was determined as:

(3) $$\;R_{\mathrm{pq}}^{\mathrm{MS}}=\frac{2F_{\mathrm{pq}}^{\mathrm{MS}}}{F_{\mathrm{pq}}^{\mathrm{MS}}+F_{\mathrm{pq}}^{\mathrm{SS}}}\;$$

where $F_{\mathrm{pq}}^{\mathrm{MS}}$ and $F_{\mathrm{pq}}^{\mathrm{SS}}$ represents frequency of contact type *pq* in the MS‐ or SS‐ dataset, respectively.

For the MS dataset, the study also calculated the frequency of each contact type *pq* when those contacts exist in one structure but break in the other:

(4)

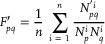

where *n* is the number of MS proteins, 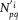 represents the overall count for all new contacts (defined as the Cα‐Cα distance in structure B is 5Å less than that in structure A) or broken contacts (defined as the Cα‐Cα distance in structure B is 5Å larger than that in structure A) in protein *i*.$\;N_{p}^{i}$ and $N_{q}^{i}$ are defined equally in Equation (2).

### HESpre Module Development

The MS data were split into training (1675 proteins), validation (180 proteins), and testing (146 proteins). To prevent overestimating the model performance due to high sequence identity, it was made sure that the sequence identity between any entry in the training set and any entry in the testing set are below 40%. For each protein in the training set, its two distance matrices were calculated to represent the structural feature of its two conformational states (structure A and B) identified in experiments. The distance matrix, denoted as *D_ij_*, is computed as:

(5) $$D_{ij}=\;1-\;{\left(1+e^{1-r_{ij}}\right)}^{-1}$$

where *r_ij_* represents the distance between the *i*th and the *j*th Cα atoms. *D_ij_* is designed as a sigmoid function to facilitate the following neural network training. *D_ij_* =  0.5 when *r_ij_* =  1 nm (contact formation), and *D_ij_* =  0 when *r_ij_* is sufficiently large (contact breaking). The deep learning model combines the two distance matrices into a L × L × 2 tensor, then converts the tensor into L × L × 64 using a convolutional layer consisting of an 8‐block Residual Convolutional Neural Network with a channel size of 64 and a kernel size of 3. Next, the model converts the L × L × 64 tensor into L × L × 1 by a fully connected layers with 64 cells, followed by adding its transpose matrix to create the symmetrical distance matrix for the transition state of this protein, $D_{ij,TS}^{predict}$. The loss function is defined as:

(6) $$\begin{array}{c} L\;=5\times \sum_{\left(i,j\right)\in unique}{\left(D_{ij,TS}^{predict}-D_{ij,TS}^{sim}\right)}^{2}+\sum_{\left(i,j\right)\in non-unique}{\left(D_{ij,TS}^{predict}-D_{ij,TS}^{sim}\right)}^{2} \end{array}$$

The same mathematic form as Equation (5) is used to calculate $D_{ij,TS}^{sim}$, while now *r_ij_* represents the averaged distance between the *i*th and the *j*th Cα atoms in the transition ensemble identified by simulations. If the transition state residues at the position of (RMSD_A, RMSD_B) in the free energy landscape, *r_ij_* was calculated locally by averaging configurations within the range of (RMSD_A±0.8Å, RMSD_B±0.8Å). Within this narrow range, each configuration was sampled with almost identical bias in metadynamics simulations, thereby causing minimal influence on the distribution. The term “unique” refers to contact formations that exist in one state but are absent in the other, whereas “non‐unique” denotes all other residue pairs. To prioritize the unique contacts, the weight ratio between the two was set at 5:1. The learning rate was set to 0.001, and Stochastic Gradient Descent (SGD) was employed as the optimization function. The model was implemented using PyTorch.

### PATHpre Model Development

The PATHpre model was built upon the HES module. For a given protein changing from its state A to state B, while the HES module aimed at predicting the pairwise residue distance matrix for the transition state, the PATHpre model simultaneously predicts the distance matrix of three special points in the transition pathway: the transition state, as well as two intermediate points between the transition state and states A/B. With these three points, the geometry of the transition pathway in the free energy landscape is almost identified. Then, other states were just interpolated among these three points. Therefore, when comparing predictions from PATHpre with simulation data (Figure S5, Supporting Information), these three points were only considered.

### Simulation with Distance Restraints

For simulations with distance restraints (taking Figure 5C as an example), first, SMOG 2.0^[^ ^36^ ^]^ was used to construct a structure‐based Hamiltonian using the open state of the MurD protein (PDB: 1e0d) as the reference. Second, all contacts in the A1‐A3 and B1 regions were removed, and they were substituted with the contacts predicted by PATHpre (*D_ij_* ≥ 0.5). The potential strength was set to 2.0 KJ mol^−1^. Ten simulated annealing simulations were carried out, spanning from 100 to 10 K within 3 × 10^6^ steps. The coarse‐grained structure can be converted to all‐atomistic representation by Pulchra, the sidechain can be further optimized by Rosetta^[^ ^49^ ^]^ or Schrödinger^[^ ^50^ ^]^ if needed. For protein depicted in Figures (5) and (6), Table S3 (Supporting Information) summarizes their MolProbity scores, along with other important metrics for structural evaluation. These metrics demonstrate that the reconstructed structures exhibit comparable quality to the input structures sourced from PDB.

### The Auto‐Encoder Model

The training data comprised 1000 structures near each state of a protein (blue dots in Figure S12A, Supporting Information), obtained from molecular dynamic simulations. The auto‐encoder was developed in Python 3.8 using PyTorch 1.8. It follows a previous work^[^ ^10a^ ^]^ which comprised two key components: an encoder and a decoder. Each of these components has a fully connected neural network with three hidden layers. The encoder takes a 3N‐dimensional vector as input, with the hidden layers containing 1000, 500, and 100 neurons, respectively. After passing through the encoder, the tensor is transformed into a 2D vector, representing a point in the latent space. Reversely, the decoder takes any vector from the latent space and ultimately recovers it into a 3N‐dimensional vector representing the coordinates of each atom. The loss function is the binary cross‐entropy, comparing the predicted structure to the actual simulated coordinates. The ReLU (Rectified Linear Unit) function was employed as the activation function. For optimization, the Adam optimizer with a learning rate of 0.001 was chosen.

### SVM and GNN

To assess the performance of the model compared to traditional machine learning methodologies, a Support Vector Machine (SVM) model^[^ ^51^ ^]^ was utilized to predict the distance matrix of the unique contacts in the transition state. The input feature comprised the distance matrix derived from the two experimentally resolved structures. The implementation leveraged the SVR (Support Vector Regression) module with a linear kernel from the Python SciPy library^[^ ^52^ ^]^ for all relevant codes.

A model based on a permutation‐equivariant graph neural network was also trained to predict the distance of the unique contact within the transition state. The flowchart is depicted in Figure S17 (Supporting Information). For a protein with two distinct structures, the node feature was the residue index 1, 2, 3… normalized by the protein length. Node *i* and node *j* are connected in the graph if residue *i* and residue *j* have formed a contact in either structure A or structure B. The edge attribute is a 2D vector (*D*_*ij*, *structure* *A*_, *D*_*ij*, *structure* *B*_) while *D_ij_* represents the pairwise distance matrix in one structure. Edge attributes were handled by utilizing a four‐layer PNAConv.^[^ ^53^ ^]^ The loss function is the MSE (Mean Square Error) loss for the distance matrix, similar to the original model. The ReLU (Rectified Linear Unit) function was employed as the activation function. For optimization, the SGD optimizer with a learning rate of 0.003 and momentum of 0.9 was chosen. The performances of these two models are shown in Table S4 (Supporting Information).

### ClustENMD and coMD

The ClustENMD algorithm was performed using ProDy.^[^ ^54^ ^]^ All parameters were kept at their default settings, except that the relaxation time was set to 3 *ps* and the number of generated conformers was set to 900, to be consistent with previous studies.^[^ ^22^ ^]^ The coMD algorithm was performed using the VMD plug‐in.^[^ ^55^ ^]^ Two different parameter settings were tested: first, using three ANM modes in the calculation to align with previous studies^[^ ^22^ ^]^; second, using the complete set of ANM modes. It is found that the number of modes did not influence the results in this system. The cycle number was set to 500, generating 3000 conformers from each input structure. All other parameters were kept at their default settings. The calculations were independently repeated six times, yielding consistent outcomes.

### Statistical Analysis

Statistical analyses of Figure 2 were performed using Python (version 3.8.10) with the pandas, seaborn, and matplotlib libraries. The results are depicted as the median, interquartile range, and the upper and lower whiskers (n = 40 082). For the rest figures, statistical analyses are not applicable.

## Conflict of Interest

The authors declare no conflict of interest.

## Author Contributions

Y.H. and H.Y. contributed equally to this work. Q.W. and F.B. designed the research. Y.H. and H.Y. performed the theoretical study. Q.W., F.B., Y.H., H.Y., M.W.L., Y.Q.Z., and Z.C.Z. analyzed the data. Q.W., F.B., Y.H., H.Y., M.W.L., and Z.C.Z. wrote the paper.

## Supporting information

Supporting Information

## Data Availability

The data that support the findings of this study are available from the corresponding author upon reasonable request.
