# Supplementary material for: Exploring Protein Conformational Changes Using a Large‐Scale Biophysical Sampling Augmented Deep Learning Strategy
Source: Adv Sci (Weinh). 2024 Oct 10;11(44):2400884. doi: 10.1002/advs.202400884 (PMC11600214; doi:10.1002/advs.202400884)
Supplement: Supplementary file 1 — Supporting Information [file ADVS-11-2400884-s001.docx]

**SI Appendix for**

**Exploring Protein Conformational Changes Using a Large-scale Biophysical Sampling Augmented Deep Learning Strategy**

Yao Hu^1,#^, Hao Yang^2,#^, Mingwei Li^1^, Zhicheng Zhong^1^, Yongqi Zhou^2^, Fang Bai^2,3,4,*^ and Qian Wang^1,*^

1. Department of Physics, University of Science and Technology of China, Hefei, Anhui, China, 230026
2. Shanghai Institute for Advanced Immunochemical Studies and School of Life Science and Technology, ShanghaiTech University, 393 Middle Huaxia Road, Shanghai, China, 201210
3. School of Information Science and Technology, ShanghaiTech University, 393 Middle Huaxia Road, Shanghai, China, 201210
4. Shanghai Clinical Research and Trial Center, Shanghai, China, 201210

#: Yao Hu and Hao Yang contributed equally

Corresponding authors:

Fang Bai (baifang@shanghaitech.edu.cn); Qian Wang (wqq@ustc.edu.cn)

**Dual basin structure-based model**

A dual basin structure-based model ^1^ was built with the following Hamiltonian:

$H=V_{bond}+V_{angle}+V_{dihedarl}+V_{nb}$ [S1]

The bonding potential $V_{bond}$ was calculated as:

$V_{bond}(r_{ij})= \epsilon_{r}\times{(r_{ij}-r_{ij}^{0})}^{2}$ [S2]

where $\epsilon_{r}=2\times{10}^{4}kJ/{nm}^{2}$. $r_{ij}^{0}$ represents the distance between residue *i* and residue *j* in the experimentally resolved structure. The dual basin angle bending potential was calculated as:

$V_{angle}(\theta_{ijk}) = \left\{ \begin{aligned} \frac{1}{2}\times\epsilon_{\theta}\times{(\theta_{ijk}-\theta_{ijk}^{01})}^{2} (\theta_{ijk}\leq\theta_{ijk}^{01}) \\ ax^{4}+bx^{2}+c (\theta_{ijk}^{01}<\theta_{ijk}<\theta_{ijk}^{02}) \\ \frac{1}{2}\times\epsilon_{\theta}\times{(\theta_{ijk}-\theta_{ijk}^{02})}^{2} (\theta_{ijk}\geq\theta_{ijk}^{02}) \end{aligned} \right.$ [S3]

where $\epsilon_{\theta}$ = 40 kJ. $\theta_{ijk}$ represents the angle formed by three continuous residues *i*, *j* and *k*. $\theta_{ijk}^{01}$ ($\theta_{ijk}^{02})$ represents the value in the experimentally resolved structure. The parameters a, b, c are chosen such that the value of $V_{angle}$, as well as its first and second derivatives are continuous at two junctions $\theta_{ijk}^{01}$, $\theta_{ijk}^{02}$. The dual basin dihedral potential $V_{dihedarl}$ followed a previous study ^1^. The non-bonded potential was calculated as

$V_{nb}=\sum_{i,j} \varepsilon_{1,2}\left[ \left( 1-e^{-\frac{\left( r_{ij}-r_{ij}^{01} \right)^{2}}{2\sigma^{2}}} \right)\left( 1-e^{-\frac{\left( r_{ij}-r_{ij}^{02} \right)^{2}}{2\sigma^{2}}} \right)\left( 1+\frac{\sigma_{rep}^{12}}{r_{ij}^{12}} \right)-1 \right]\Delta_{ij}+\sum_{i,j} \varepsilon\left( \frac{\sigma_{rep}^{12}}{r_{ij}^{12}} \right){(1-\Delta}_{ij})$ [S4]

$r_{ij}^{01}$ and $r_{ij}^{02}$ represent the distance between residue *i* and *j* in two folding topologies, respectively. $\Delta_{ij} =1$ if residues *i* and *j* form a contact in either topology; otherwise $\Delta_{ij} =0$. $\varepsilon_{1,2}$ represent the solvent mediated interactions of the contacts. $\varepsilon=\varepsilon_{1,2}$ = 1.0 kJ/mol. $\sigma$ = 0.05 nm. $\sigma_{rep}$= 0.4 nm.

1. Lin, X.; Eddy, N. R.; Noel, J. K.; Whitford, P. C.; Wang, Q.; Ma, J.; Onuchic, J. N., Order and disorder control the functional rearrangement of influenza hemagglutinin. *Proceedings of the National Academy of Sciences of the United States of America* **2014,** *111* (33), 12049-12054.

**Figure S1.** Distribution of the MolProbity score percentile for the reverse-mapped structures along the transition pathway.


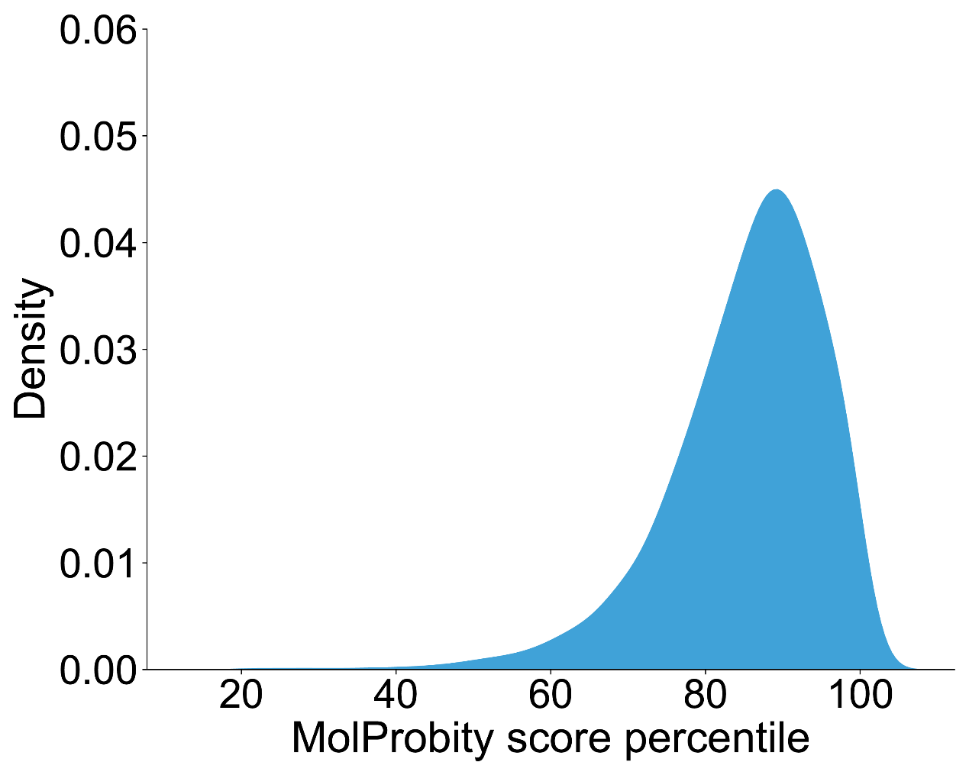


Figure S2. Correlation between the Euclidean distance matrix and the sequence distance matrix ($|i-j|$) for the contacts used to evaluate our model.


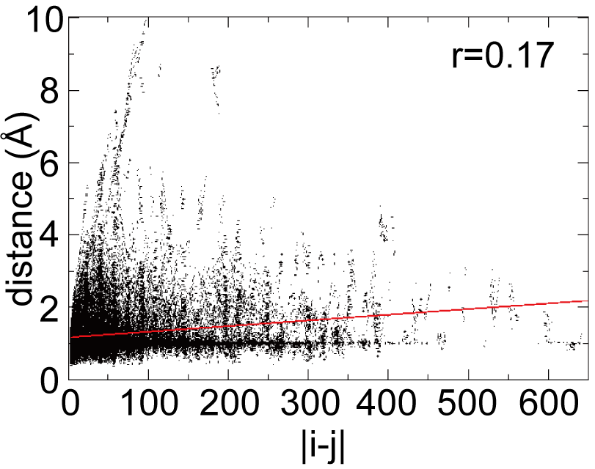


**Figure S3.** Comparison between our prediction and the simulation data in different subgroups of the testing set. The subgroups are divided based on the sequence length, the structural characteristics (fold switching or no fold-switching), or the RMSD between two experimentally resolved structures. Each panel represents a different subgroup. *r* represents the Pearson correlation coefficient. MAE represents the mean absolute error. $D_{ij}$ represents the pairwise distance matrix for “unique” contact formations (only exist in one experimentally resolved structure but not in the other). Each point describes one $D_{ij}$ in one protein. $D_{ij}$ from all proteins are plotted together in the figure.

**
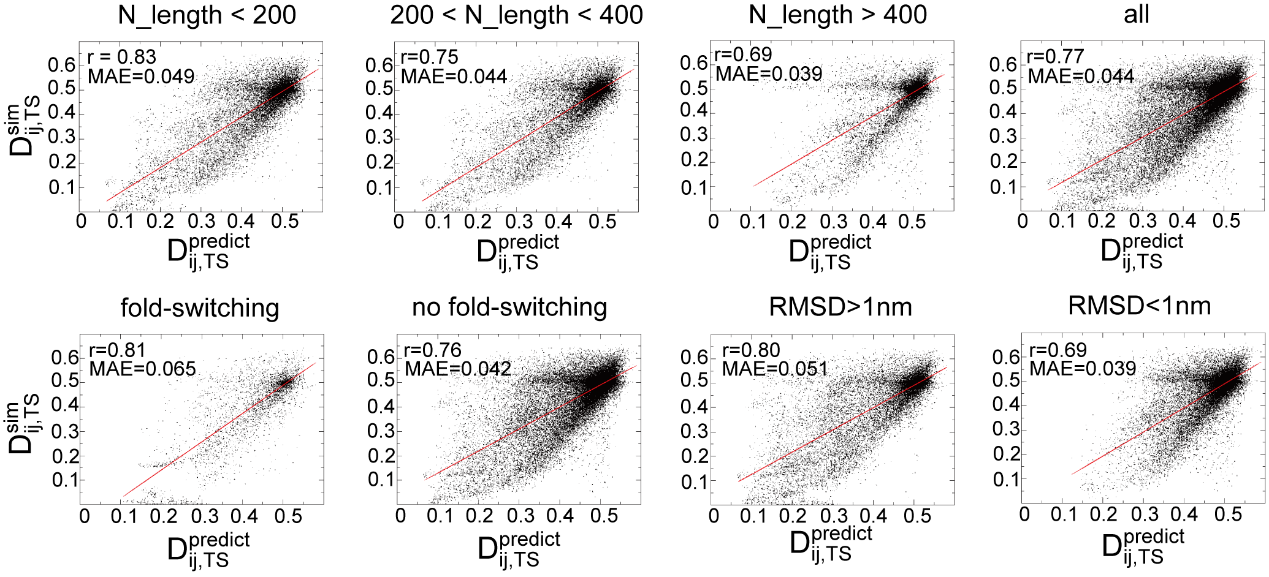
**

**Figure S4.** Comparison between contact maps averaged by all structures in the transition state predicted by HESpre (“pred”) with that directly extracted from metadynamics simulations (“sim”) for 8 different proteins. The contact map is averaged by all structures in the transition state. A contact is defined as a residue-residue distance less than 10Å, averaged across various structures within the transition ensemble. N represent the number of amino acids. Accuracy represent the ratio of correct predictions within those “unique” contacts (only exist in one experimentally resolved structure but not in the other).

**
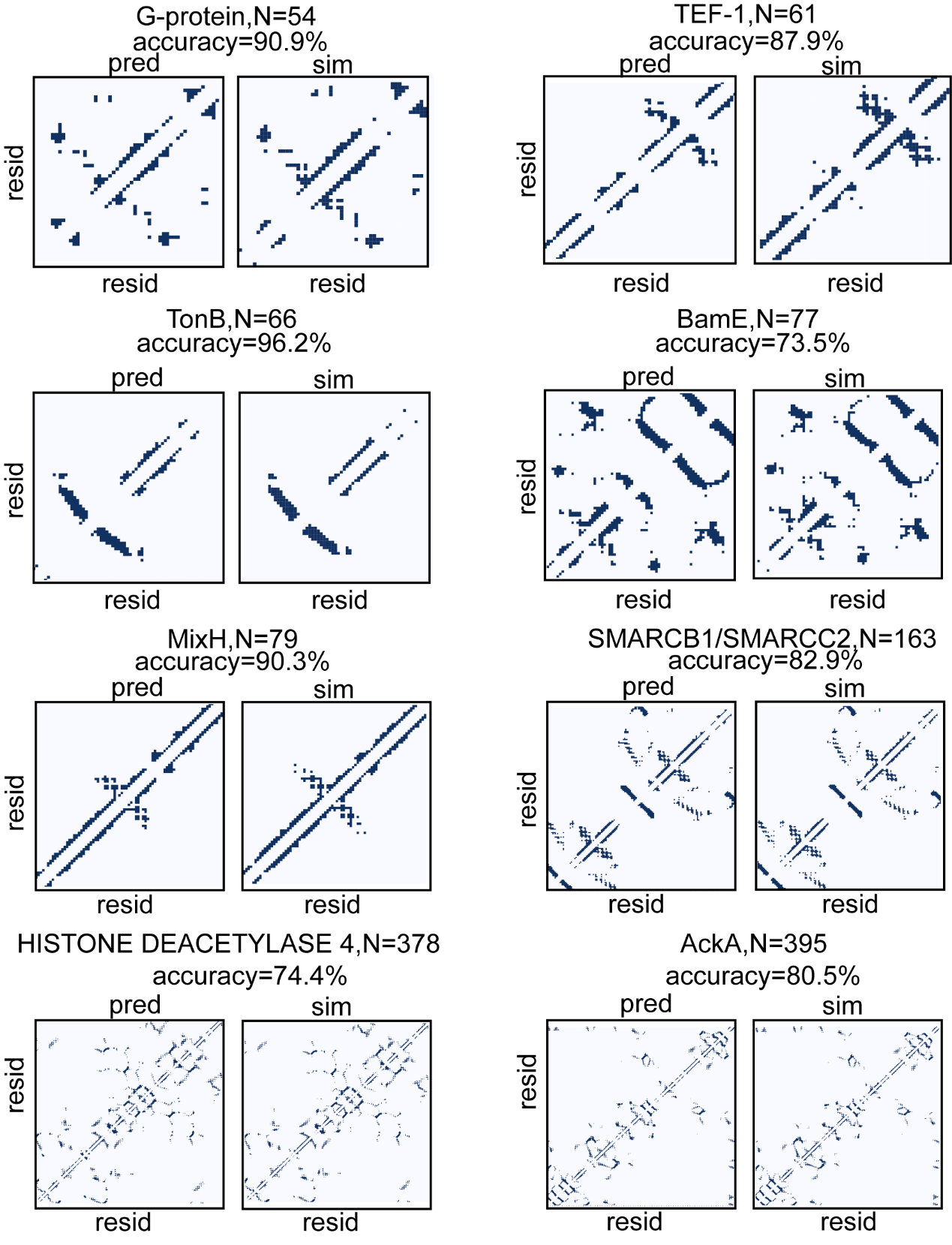
**

**Figure S5.** Comparison between our prediction and the simulation data in different states along the transition pathway. For a protein changing from state A to state B, state 3 is the transition state. State 2 is the middle point between the transition state and the state A. State 4 is the middle point between the transition state and the state B. *r* represents the Pearson correlation coefficient. MAE represents the mean absolute error. $D_{ij}$ represents the pairwise distance matrix for “unique” contact formations (only exist in one experimentally resolved structure but not in the other). Each point describes one $D_{ij}$ in one protein. $D_{ij}$ from all proteins are plotted together in the figure.

**
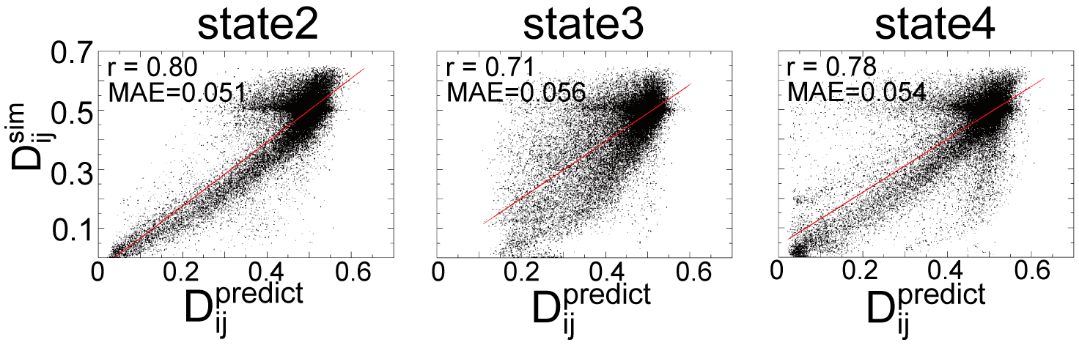
**

Figure S6. Generated conformer ensembles by our model for (A) protein HIV-1 protease and (B) protein PGK, projected onto a subspace spanned by experimental PCs. The initial structures used in our model are represented by yellow asterisks. The distribution of generated structures is displayed by contour plots. Experimentally resolved structures, represented by red dots, are searched in the Protein Data Bank with sequence identity greater than 90%. There are 26 structures for PGK and 644 structures for HIV-1 protease.


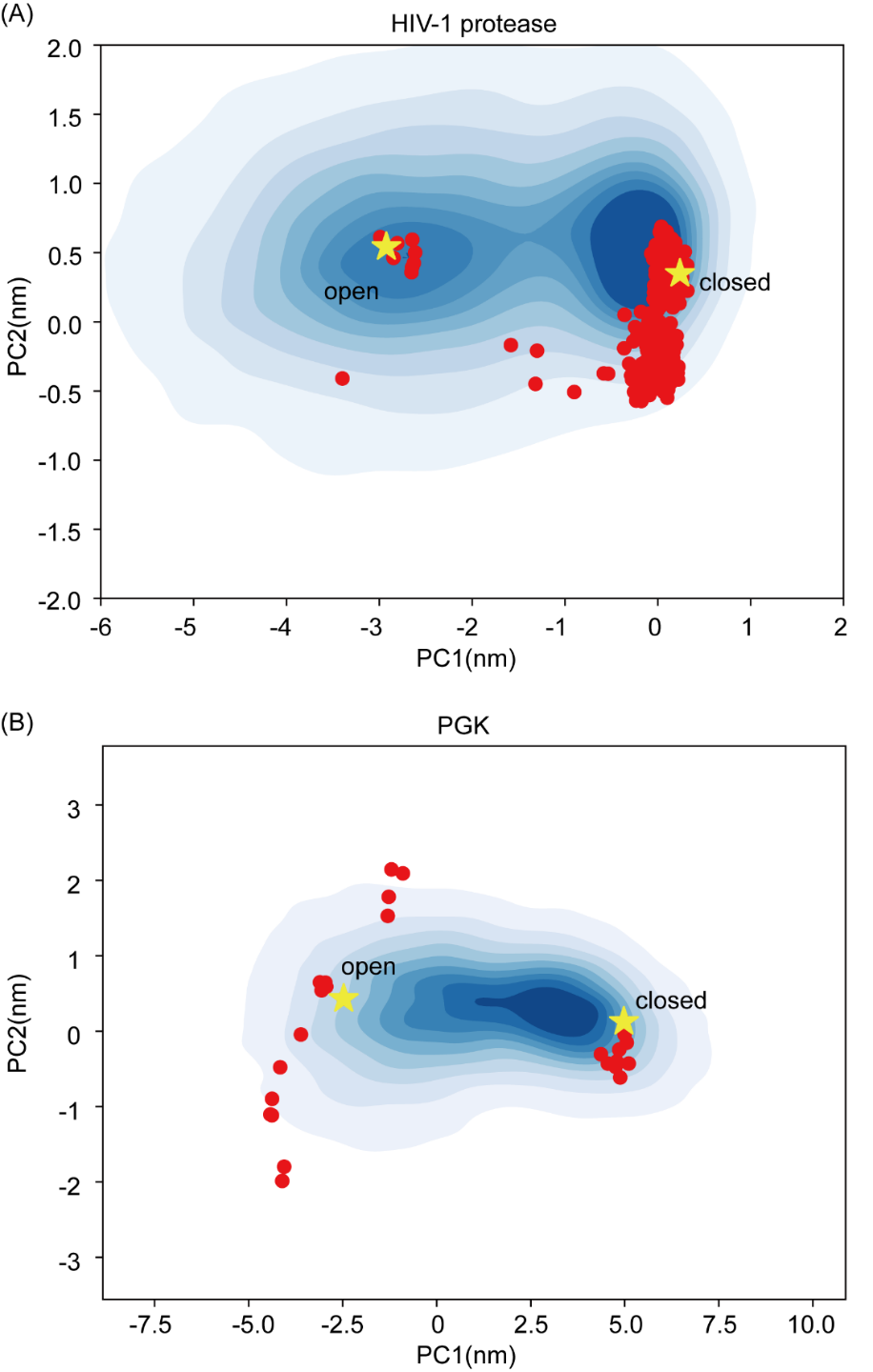


Figure S7. Generated conformer ensemble (blue and red dots) by ENM-based methods. (A) 30S ribosomal protein S7 predicted by the ClustENMD algorithm; (B) protein RfaH predicted by the ClustENMD algorithm; and (C) protein RfaH predicted by the coMD algorithm. Conformers are projected onto the free energy landscape from metadynamics simulations.


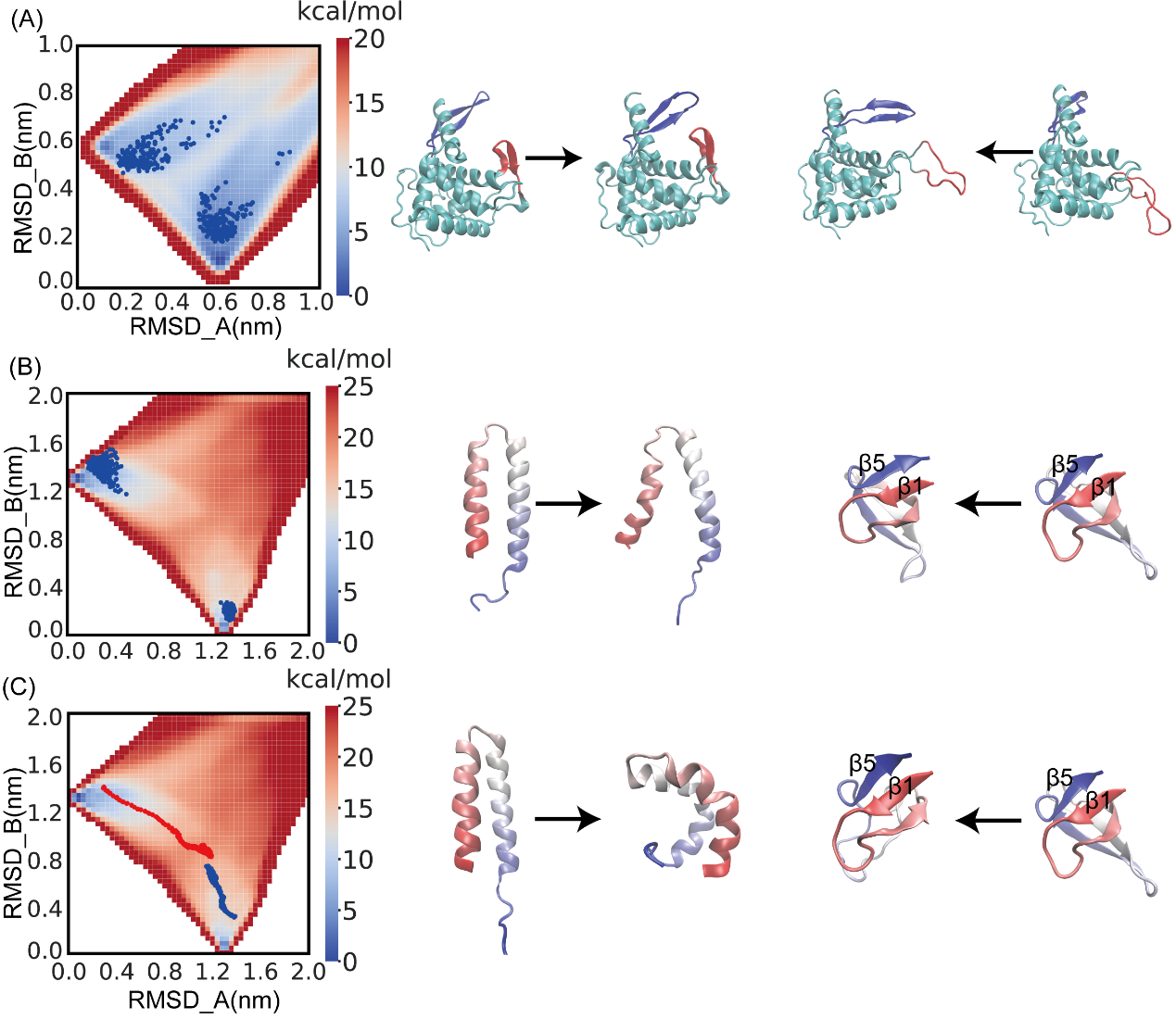


Figure S8. Projection of three generated states by PATHpre onto the free energy landscape for (A) protein RfaH and (B) protein MurD. We have generated 10 structures for each state. Each yellow point represents a generated structure. The red asterisks represent the averaged position for all structures belonging to the same state. Region A1-A3 and B1 follow the same definition as Figure 5C in the main text.


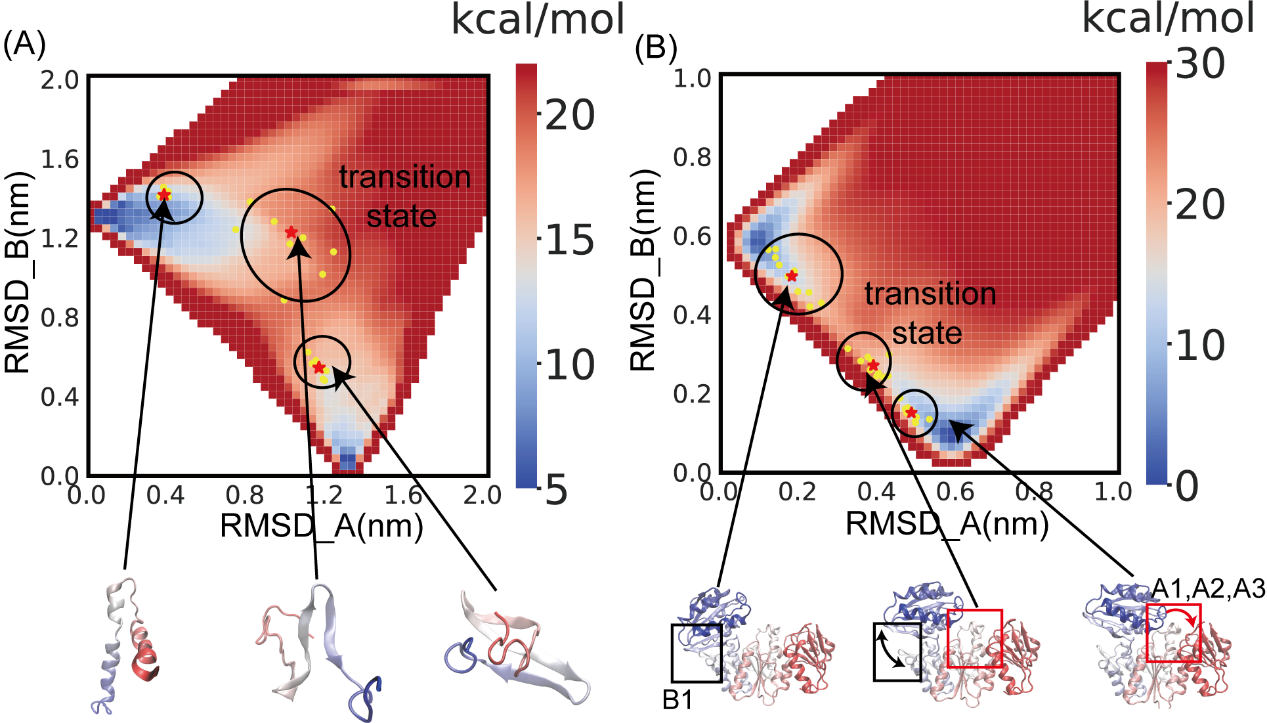


Figure S9. Replication of the simulation shown in Figure 6C in the main text.


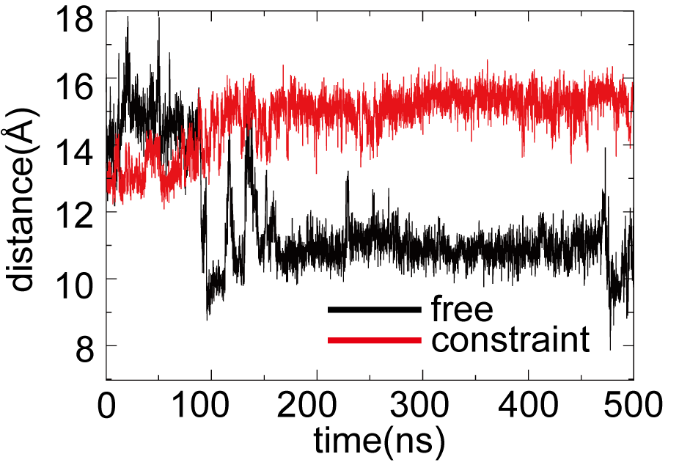


Figure S10. Free energy landscape of proteins exhibiting multiple intermediate metastable states: (A) adenylate kinase, PDB ID: 4ake and 7apu; (B) Caspase recruitment domain-containing protein 4, PDB ID: 2nsn and 2b1w; (C) SOSS complex subunit B1, PDB ID: 5d8f and 4owt; (D) Glucagon receptor, PDB ID: 5yqz and 5xf1; (E) Stromal interaction molecule 1, PDB ID: 6yel and 4o9b. Personalized collective variables were designed for each protein to distinguish these intermediate metastable states, which are represented by black asterisks.


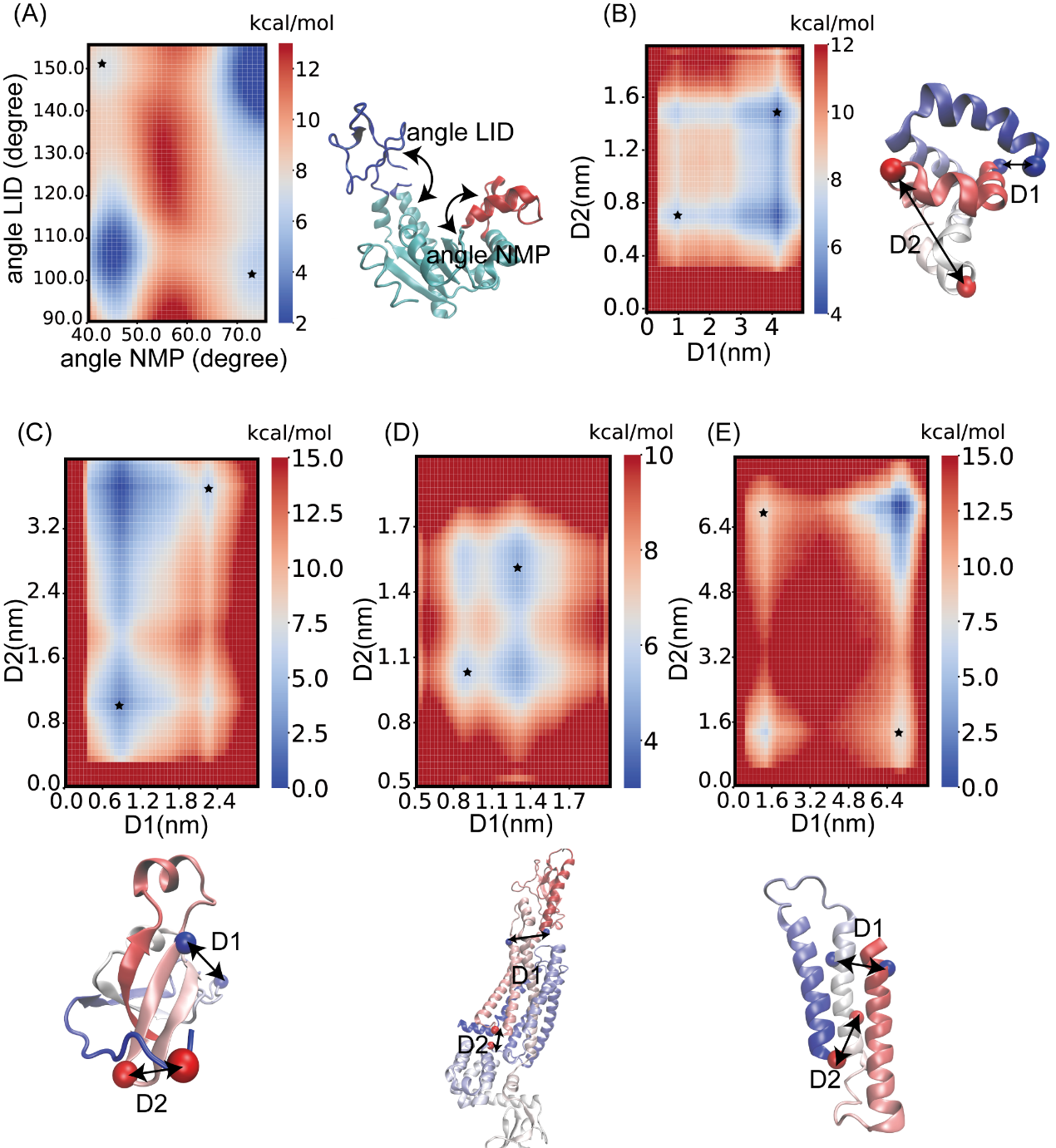


Figure S11. Free energy landscape of proteins exhibiting multiple intermediate metastable conformations: (A) Small archaeal modifier protein 2, PDB ID: 2l32 and 4hrs; (B) AMA 1, PDB ID: 3zle and 3zld; (C) Probable GTPase engC, PDB ID: 4a2i and 2rcn; (D) RPRD1B, PDB ID: 4hfg and 4q96; (E) RNA polymerase, PDB ID: 6ntv and 7alp. The collective variables are RMSD_A and RMSD_B, representing the root-mean-square deviation of simulated structures with respect to experimentally resolved structure A and structure B, respectively. Only Cα atoms are used in the calculations. The intermediate metastable states are represented by black asterisks.


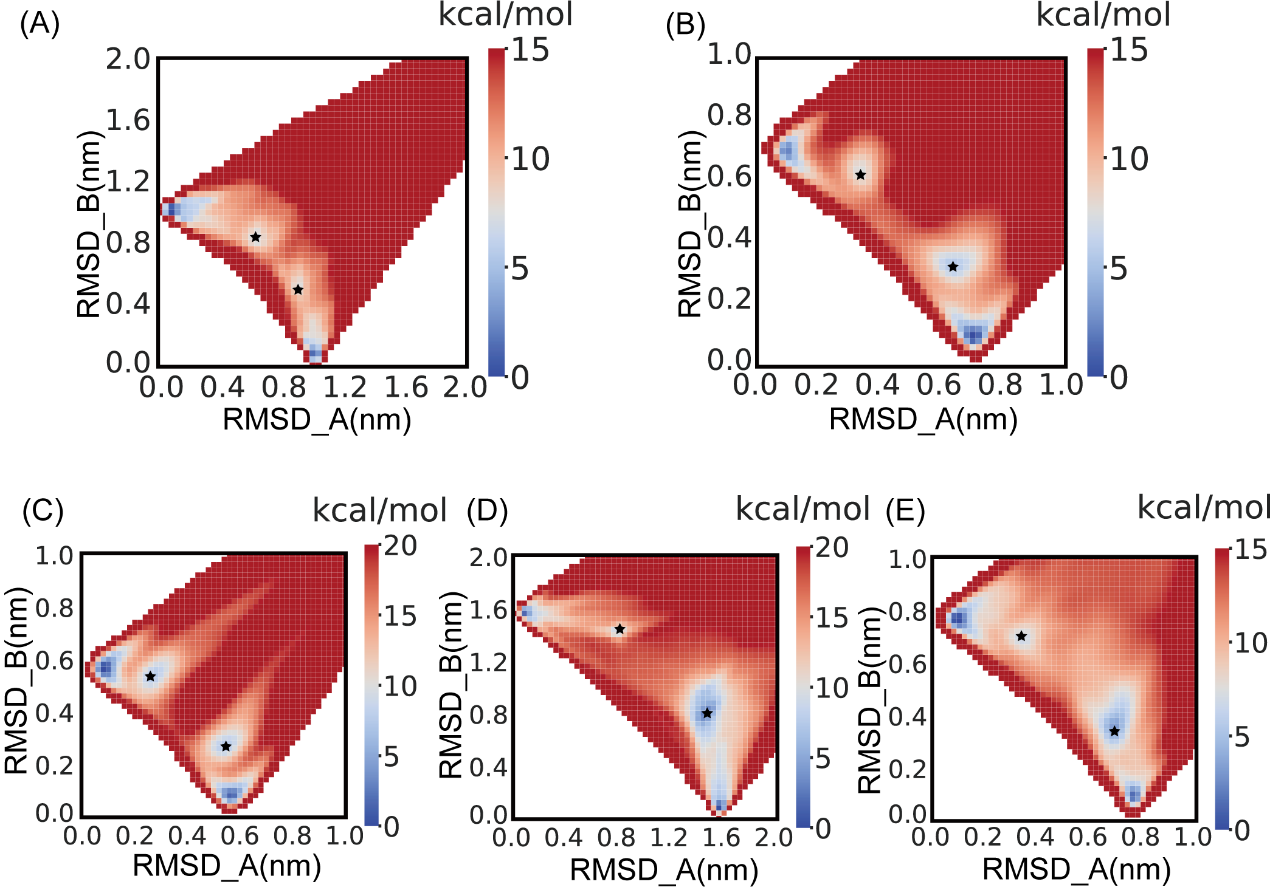


Figure S12. Examine whether the transition pathway is linear in the latent space for (A) the protein adenylate kinase (AdK) and (B) the protein RfaH. In Panel (A), blue dots represent the structures near the two conformational states (training data). Black dots represent the actual transition pathway identified in simulations. Orange dots represent the linear interpolation in the latent space. Panel (B) shows the folding order of several beta sheets along the transition pathway (index from 0 to 100) calculated by linear interpolation in the latent space. Q_contact represents the fraction of the contact formations. The result indicates that the contracts between β1 and β5 (red line) form in the early stage, which is inconsistent with multiple previous studies.

**
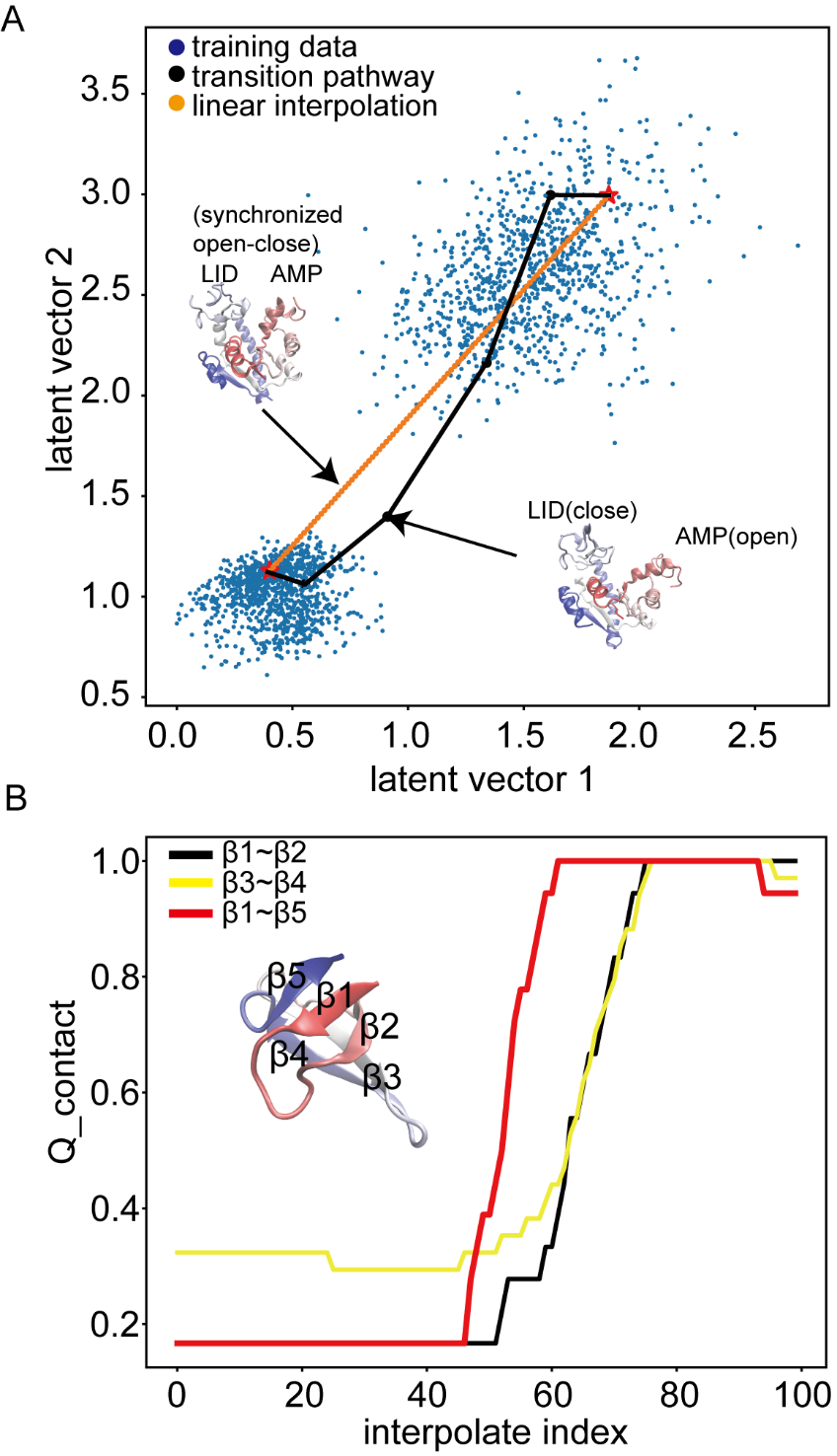
**

Figure S13. Comparison between our prediction and the simulation data for protein transitions with RMSD between 0.2 and 0.5 nm). *r* represents the Pearson correlation coefficient. $D_{ij}$ represents the pairwise distance matrix for unique contact formations in the transition state.


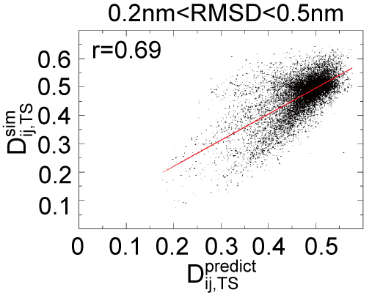


Figure S14. Violin plots of the RMSD (Root Mean Square Deviation) and differences in *R_g_* (Radius of Gyration) between two representative conformations for Category I and II types of MS proteins. Category I is depicted in red, while Category II is shown in blue.


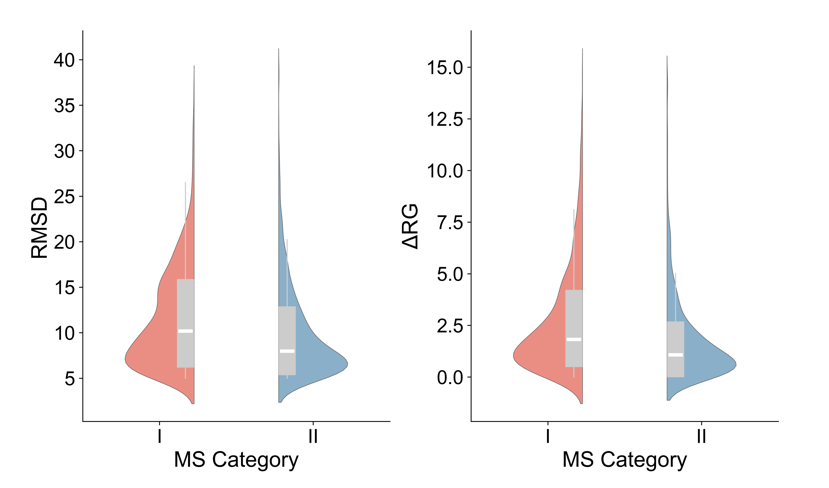


Figure S15. Free energy landscape of protein TEF-1 (A-D) and Nuclear receptor corepressor 2 (E-H) with different simulation lengths and different structures initiating the simulation.


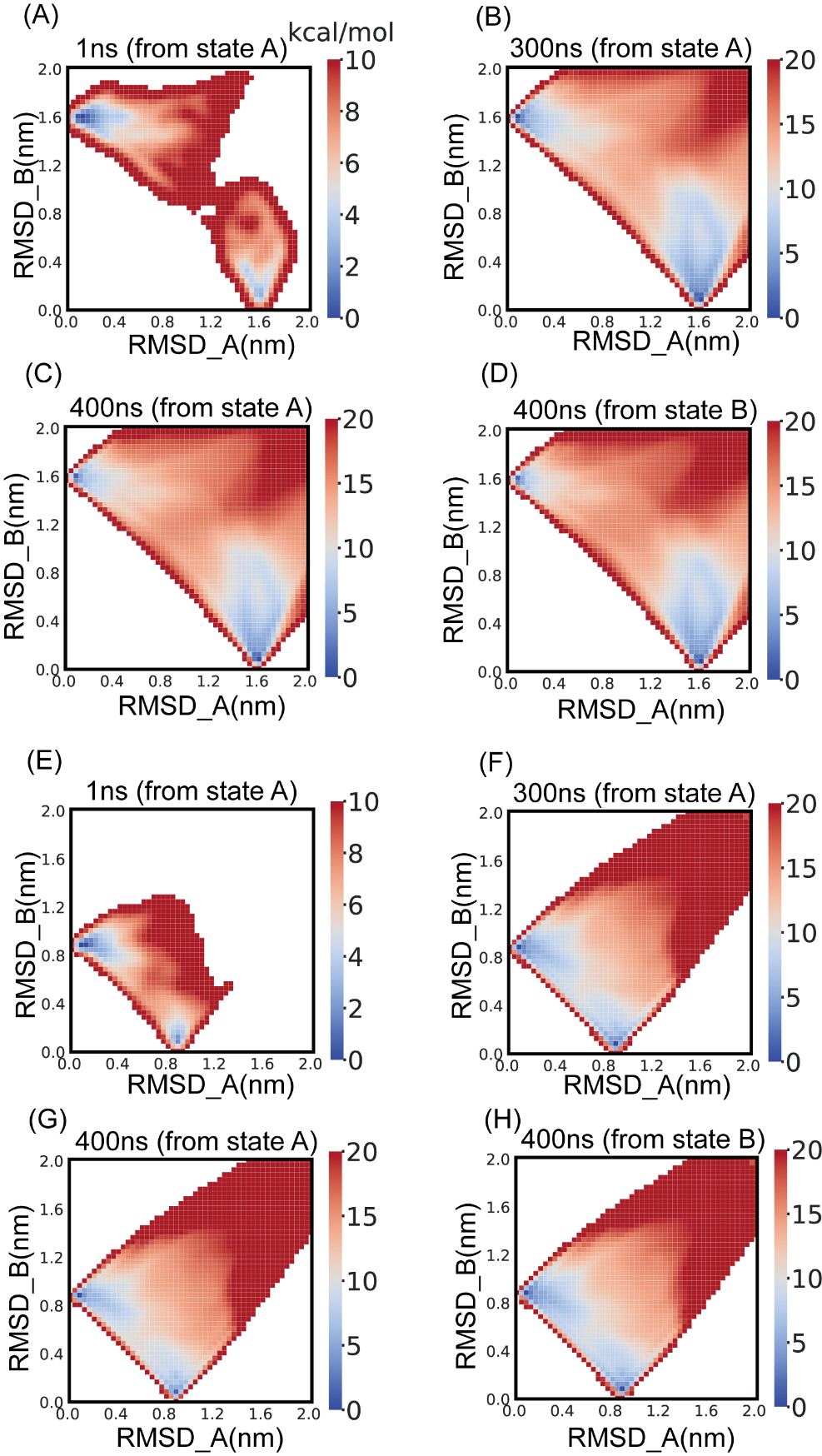


Figure S16. Time evolution of the collective variables for (A) protein RNA polymerase sigma factor RpoD and (B) protein TEF-1. Only a subset of the trajectories is shown as representative. Blue box highlights the visit to the two distinct conformational states of the protein. The right panel represents the free energy landscape.


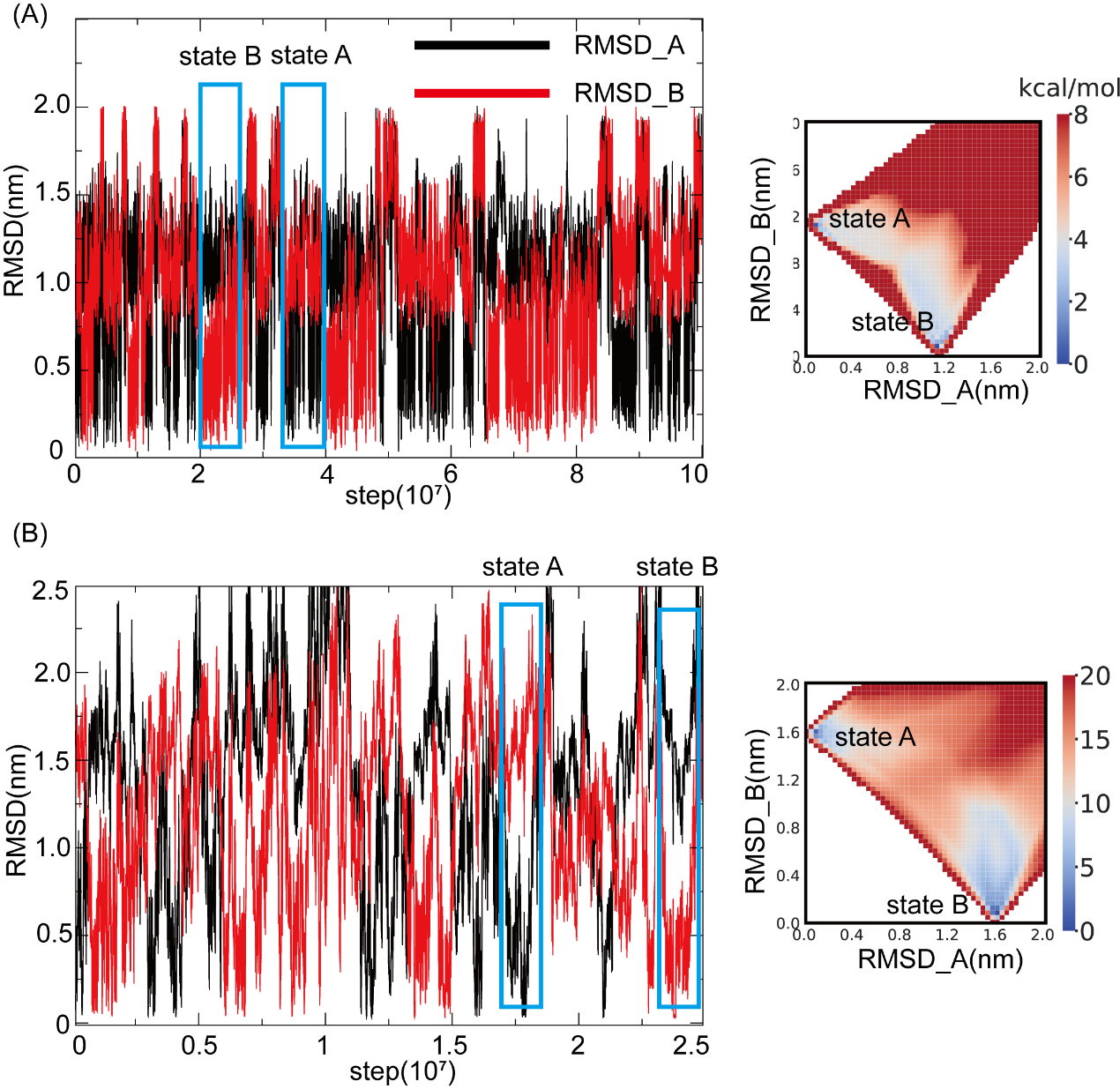


Figure S17. Architecture of the permutation-equivariant graph neural network. More details can be found in the methods section.


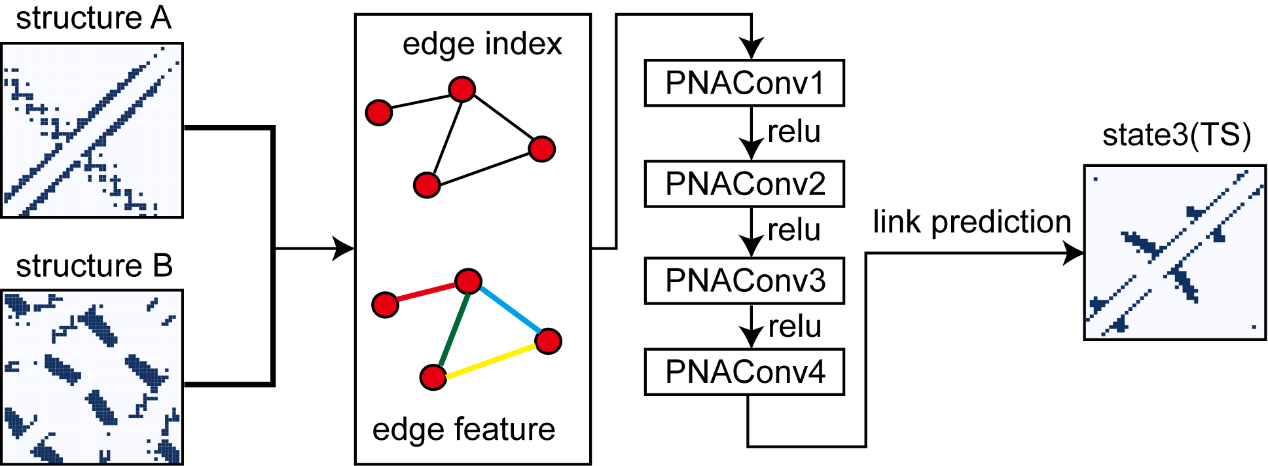


**Supplementary Table S1**. Frequency ratio of each contact type in the Multi-State dataset versus the corresponding frequency in the Single-State dataset.

| **Rank** | **Residue Pair** | | **Frequency Ratio** | **Rank** | **Residue Pair** | **Frequency Ratio** |
| --- | --- | --- | --- | --- | --- | --- |
| 1 | ARG-GLU | 1.126 | | 106 | ARG-TRP | 1.050 |
| 2 | GLN-GLU | 1.125 | | 107 | GLY-VAL | 1.050 |
| 3 | GLN-GLN | 1.121 | | 108 | GLN-GLY | 1.050 |
| 4 | ARG-ILE | 1.109 | | 109 | ASN-PRO | 1.050 |
| 5 | GLU-LEU | 1.106 | | 110 | PRO-VAL | 1.050 |
| 6 | GLU-GLU | 1.106 | | 111 | ASP-MET | 1.049 |
| 7 | ASP-GLU | 1.105 | | 112 | ASP-LYS | 1.049 |
| 8 | ARG-THR | 1.105 | | 113 | ARG-HIS | 1.048 |
| 9 | ARG-LYS | 1.102 | | 114 | GLU-TRP | 1.048 |
| 10 | GLN-LEU | 1.101 | | 115 | ASN-VAL | 1.048 |
| 11 | ARG-ARG | 1.100 | | 116 | SER-SER | 1.048 |
| 12 | GLU-THR | 1.099 | | 117 | LEU-THR | 1.046 |
| 13 | ARG-ASN | 1.099 | | 118 | ASN-SER | 1.046 |
| 14 | GLU-ILE | 1.098 | | 119 | ILE-THR | 1.046 |
| 15 | GLU-PHE | 1.097 | | 120 | ASN-MET | 1.046 |
| 16 | ILE-LYS | 1.096 | | 121 | GLY-ILE | 1.045 |
| 17 | GLU-PRO | 1.095 | | 122 | PHE-TYR | 1.045 |
| 18 | ARG-VAL | 1.095 | | 123 | SER-THR | 1.045 |
| 19 | ARG-LEU | 1.095 | | 124 | GLY-LEU | 1.044 |
| 20 | GLN-LYS | 1.094 | | 125 | ASN-TYR | 1.042 |
| 21 | LYS-PHE | 1.093 | | 126 | PRO-SER | 1.042 |
| 22 | ARG-SER | 1.092 | | 127 | VAL-VAL | 1.041 |
| 23 | ASP-ILE | 1.092 | | 128 | ILE-SER | 1.041 |
| 24 | ALA-GLU | 1.091 | | 129 | HIS-PRO | 1.041 |
| 25 | GLU-VAL | 1.089 | | 130 | TRP-VAL | 1.041 |
| 26 | ALA-LYS | 1.089 | | 131 | ASP-GLY | 1.039 |
| 27 | ARG-MET | 1.088 | | 132 | ASN-HIS | 1.039 |
| 28 | ARG-TYR | 1.086 | | 133 | HIS-PHE | 1.038 |
| 29 | ASP-VAL | 1.085 | | 134 | GLY-SER | 1.038 |
| 30 | ARG-PRO | 1.084 | | 135 | CYS-PHE | 1.038 |
| 31 | LYS-LYS | 1.084 | | 136 | CYS-LEU | 1.037 |
| 32 | GLU-SER | 1.083 | | 137 | HIS-THR | 1.037 |
| 33 | ASN-GLU | 1.080 | | 138 | GLU-MET | 1.037 |
| 34 | GLU-TYR | 1.080 | | 139 | CYS-GLU | 1.035 |
| 35 | ALA-ARG | 1.079 | | 140 | ALA-SER | 1.035 |
| 36 | LEU-LYS | 1.079 | | 141 | GLN-MET | 1.034 |
| 37 | ARG-PHE | 1.078 | | 142 | GLY-PHE | 1.033 |
| 38 | LYS-VAL | 1.078 | | 143 | PHE-PRO | 1.032 |
| 39 | ASP-GLN | 1.077 | | 144 | THR-TYR | 1.032 |
| 40 | ASP-PHE | 1.074 | | 145 | CYS-VAL | 1.031 |
| 41 | ASP-TYR | 1.074 | | 146 | HIS-SER | 1.031 |
| 42 | ASP-SER | 1.074 | | 147 | GLN-PRO | 1.031 |
| 43 | GLU-GLY | 1.073 | | 148 | ALA-VAL | 1.031 |
| 44 | ASP-THR | 1.073 | | 149 | MET-PHE | 1.029 |
| 45 | GLN-VAL | 1.073 | | 150 | ILE-MET | 1.029 |
| 46 | ASP-LEU | 1.072 | | 151 | GLY-TYR | 1.028 |
| 47 | LYS-PRO | 1.071 | | 152 | MET-THR | 1.028 |
| 48 | ALA-GLN | 1.071 | | 153 | ALA-GLY | 1.027 |
| 49 | HIS-LYS | 1.071 | | 154 | GLY-THR | 1.027 |
| 50 | ALA-THR | 1.070 | | 155 | ILE-ILE | 1.027 |
| 51 | ASN-LYS | 1.069 | | 156 | PRO-TYR | 1.026 |
| 52 | LEU-SER | 1.069 | | 157 | TRP-TYR | 1.024 |
| 53 | GLN-HIS | 1.069 | | 158 | GLY-PRO | 1.024 |
| 54 | LYS-THR | 1.068 | | 159 | GLY-HIS | 1.022 |
| 55 | GLN-PHE | 1.068 | | 160 | MET-PRO | 1.021 |
| 56 | GLU-LYS | 1.068 | | 161 | LYS-MET | 1.018 |
| 57 | ASN-GLN | 1.067 | | 162 | MET-VAL | 1.018 |
| 58 | ALA-ASN | 1.067 | | 163 | ALA-MET | 1.017 |
| 59 | ARG-ASP | 1.067 | | 164 | PHE-THR | 1.016 |
| 60 | GLN-TYR | 1.067 | | 165 | MET-MET | 1.014 |
| 61 | ASN-ILE | 1.066 | | 166 | ASP-HIS | 1.013 |
| 62 | GLY-LYS | 1.066 | | 167 | HIS-HIS | 1.013 |
| 63 | TYR-VAL | 1.065 | | 168 | LEU-MET | 1.012 |
| 64 | PHE-SER | 1.065 | | 169 | ASN-GLY | 1.012 |
| 65 | LEU-LEU | 1.065 | | 170 | SER-TYR | 1.011 |
| 66 | HIS-ILE | 1.065 | | 171 | CYS-THR | 1.010 |
| 67 | ARG-GLY | 1.065 | | 172 | MET-TRP | 1.007 |
| 68 | ASN-LEU | 1.064 | | 173 | CYS-GLN | 1.006 |
| 69 | GLN-THR | 1.064 | | 174 | ALA-HIS | 1.005 |
| 70 | SER-VAL | 1.064 | | 175 | ALA-ALA | 1.004 |
| 71 | ASN-THR | 1.063 | | 176 | MET-SER | 1.004 |
| 72 | ILE-LEU | 1.063 | | 177 | TRP-TRP | 1.003 |
| 73 | GLN-SER | 1.062 | | 178 | ILE-TRP | 1.001 |
| 74 | THR-THR | 1.062 | | 179 | ALA-TRP | 0.997 |
| 75 | ALA-ASP | 1.062 | | 180 | PRO-PRO | 0.997 |
| 76 | LEU-TYR | 1.060 | | 181 | ASN-ASN | 0.996 |
| 77 | PHE-VAL | 1.060 | | 182 | ASP-TRP | 0.995 |
| 78 | ILE-TYR | 1.059 | | 183 | CYS-ILE | 0.994 |
| 79 | ALA-TYR | 1.059 | | 184 | LEU-TRP | 0.994 |
| 80 | LYS-SER | 1.059 | | 185 | HIS-LEU | 0.993 |
| 81 | GLU-HIS | 1.059 | | 186 | PHE-TRP | 0.992 |
| 82 | LEU-PRO | 1.058 | | 187 | GLY-MET | 0.986 |
| 83 | CYS-LYS | 1.058 | | 188 | LYS-TRP | 0.985 |
| 84 | THR-VAL | 1.058 | | 189 | CYS-TYR | 0.984 |
| 85 | GLN-ILE | 1.058 | | 190 | ASP-CYS | 0.984 |
| 86 | HIS-VAL | 1.057 | | 191 | SER-TRP | 0.984 |
| 87 | LEU-PHE | 1.057 | | 192 | GLN-TRP | 0.983 |
| 88 | LYS-TYR | 1.057 | | 193 | ASN-CYS | 0.983 |
| 89 | ALA-PRO | 1.056 | | 194 | TYR-TYR | 0.981 |
| 90 | ASN-PHE | 1.056 | | 195 | ALA-CYS | 0.980 |
| 91 | ILE-VAL | 1.056 | | 196 | PHE-PHE | 0.979 |
| 92 | LEU-VAL | 1.055 | | 197 | GLY-TRP | 0.976 |
| 93 | ILE-PHE | 1.055 | | 198 | ASN-TRP | 0.976 |
| 94 | HIS-TYR | 1.054 | | 199 | CYS-TRP | 0.976 |
| 95 | ARG-GLN | 1.054 | | 200 | GLY-GLY | 0.971 |
| 96 | MET-TYR | 1.054 | | 201 | CYS-PRO | 0.964 |
| 97 | ALA-LEU | 1.054 | | 202 | CYS-GLY | 0.959 |
| 98 | ASN-ASP | 1.054 | | 203 | HIS-TRP | 0.958 |
| 99 | ILE-PRO | 1.052 | | 204 | CYS-SER | 0.955 |
| 100 | PRO-THR | 1.052 | | 205 | THR-TRP | 0.954 |
| 101 | ASP-PRO | 1.052 | | 206 | CYS-MET | 0.953 |
| 102 | ALA-PHE | 1.052 | | 207 | HIS-MET | 0.937 |
| 103 | ALA-ILE | 1.051 | | 208 | CYS-HIS | 0.935 |
| 104 | ARG-CYS | 1.051 | | 209 | CYS-CYS | 0.925 |
| 105 | ASP-ASP | 1.050 | | 210 | PRO-TRP | 0.921 |

**Supplementary Table S2.** Frequency of newly formed and broken residue contacts along the transition pathways of conformational changes in the Multi-State dataset.

| **Pair** | **new contact**  **frequency** | | **broken contact**  **frequency** | **Pair** | **new contact**  **frequency** | **broken contact frequency** |
| --- | --- | --- | --- | --- | --- | --- |
| GLN-LYS | 0.1388 | 0.1283 | | GLY-LEU | 0.1250 | 0.1197 |
| ASP-SER | 0.1379 | 0.1239 | | ILE-PRO | 0.1247 | 0.1193 |
| ASP-LYS | 0.1378 | 0.1272 | | THR-TYR | 0.1246 | 0.1187 |
| ARG-GLU | 0.1378 | 0.1271 | | ASN-PHE | 0.1244 | 0.1211 |
| ARG-LYS | 0.1376 | 0.1268 | | ALA-GLY | 0.1243 | 0.1204 |
| LYS-SER | 0.1376 | 0.1270 | | GLY-PHE | 0.1241 | 0.1182 |
| ASN-GLU | 0.1375 | 0.1268 | | GLY-ILE | 0.1241 | 0.1151 |
| GLN-SER | 0.1374 | 0.1258 | | HIS-THR | 0.1240 | 0.1150 |
| GLU-SER | 0.1373 | 0.1291 | | ALA-PHE | 0.1236 | 0.1200 |
| ARG-SER | 0.1373 | 0.1254 | | GLY-VAL | 0.1236 | 0.1154 |
| ASN-SER | 0.1372 | 0.1241 | | ALA-TYR | 0.1233 | 0.1177 |
| ASN-LYS | 0.1370 | 0.1270 | | GLY-TYR | 0.1233 | 0.1153 |
| ARG-ASN | 0.1367 | 0.1261 | | PRO-VAL | 0.1233 | 0.1145 |
| ASP-GLU | 0.1364 | 0.1281 | | TYR-VAL | 0.1231 | 0.1127 |
| GLU-LYS | 0.1361 | 0.1282 | | HIS-ILE | 0.1230 | 0.1108 |
| GLN-GLU | 0.1359 | 0.1286 | | THR-VAL | 0.1227 | 0.1154 |
| ARG-ASP | 0.1358 | 0.1266 | | LEU-PHE | 0.1227 | 0.1217 |
| SER-THR | 0.1357 | 0.1200 | | LEU-PRO | 0.1225 | 0.1191 |
| ARG-THR | 0.1351 | 0.1232 | | ILE-PHE | 0.1221 | 0.1170 |
| ASN-ASP | 0.1349 | 0.1277 | | PHE-VAL | 0.1218 | 0.1181 |
| LYS-THR | 0.1348 | 0.1244 | | PHE-TYR | 0.1216 | 0.1167 |
| ASN-GLN | 0.1344 | 0.1261 | | ILE-TYR | 0.1215 | 0.1144 |
| ASP-GLN | 0.1344 | 0.1266 | | LEU-TYR | 0.1212 | 0.1171 |
| GLU-THR | 0.1343 | 0.1261 | | GLY-HIS | 0.1210 | 0.1124 |
| GLY-LYS | 0.1336 | 0.1252 | | HIS-TYR | 0.1207 | 0.1094 |
| ASP-THR | 0.1331 | 0.1233 | | PRO-TYR | 0.1206 | 0.1178 |
| GLN-THR | 0.1331 | 0.1224 | | ALA-HIS | 0.1202 | 0.1146 |
| GLU-ILE | 0.1329 | 0.1235 | | HIS-LEU | 0.1184 | 0.1131 |
| ASN-THR | 0.1328 | 0.1232 | | LYS-MET | 0.1176 | 0.1134 |
| GLU-GLY | 0.1326 | 0.1272 | | MET-SER | 0.1172 | 0.1118 |
| ALA-GLU | 0.1322 | 0.1228 | | HIS-PRO | 0.1172 | 0.1111 |
| ARG-GLN | 0.1320 | 0.1264 | | ASN-MET | 0.1167 | 0.1116 |
| ALA-LYS | 0.1319 | 0.1218 | | HIS-VAL | 0.1164 | 0.1083 |
| GLU-PRO | 0.1319 | 0.1261 | | GLU-MET | 0.1152 | 0.1158 |
| ASP-GLY | 0.1319 | 0.1231 | | MET-THR | 0.1146 | 0.1102 |
| ALA-ASP | 0.1318 | 0.1218 | | HIS-PHE | 0.1145 | 0.1110 |
| LEU-LYS | 0.1318 | 0.1239 | | ARG-MET | 0.1144 | 0.1102 |
| ASN-GLY | 0.1317 | 0.1209 | | MET-PRO | 0.1140 | 0.1085 |
| GLN-GLY | 0.1317 | 0.1242 | | GLN-MET | 0.1138 | 0.1099 |
| ALA-SER | 0.1313 | 0.1210 | | MET-VAL | 0.1134 | 0.1087 |
| LYS-PRO | 0.1312 | 0.1245 | | ASP-MET | 0.1132 | 0.1096 |
| ILE-LYS | 0.1311 | 0.1208 | | ASN-TRP | 0.1129 | 0.0964 |
| ARG-VAL | 0.1310 | 0.1197 | | LYS-TRP | 0.1123 | 0.0989 |
| ILE-SER | 0.1309 | 0.1210 | | HIS-MET | 0.1121 | 0.1067 |
| SER-VAL | 0.1309 | 0.1163 | | LEU-MET | 0.1119 | 0.1101 |
| GLY-SER | 0.1308 | 0.1204 | | ILE-MET | 0.1117 | 0.1084 |
| ALA-ASN | 0.1305 | 0.1214 | | ALA-MET | 0.1114 | 0.1093 |
| ARG-PRO | 0.1305 | 0.1246 | | GLY-MET | 0.1114 | 0.1067 |
| GLU-PHE | 0.1304 | 0.1250 | | ARG-TRP | 0.1112 | 0.0979 |
| GLN-VAL | 0.1304 | 0.1200 | | THR-TRP | 0.1109 | 0.0953 |
| ASN-LEU | 0.1303 | 0.1213 | | SER-TRP | 0.1105 | 0.0951 |
| GLU-LEU | 0.1302 | 0.1258 | | GLN-TRP | 0.1100 | 0.0995 |
| GLU-VAL | 0.1302 | 0.1218 | | GLU-TRP | 0.1100 | 0.0989 |
| GLN-PRO | 0.1301 | 0.1247 | | ILE-TRP | 0.1095 | 0.0949 |
| ALA-GLN | 0.1301 | 0.1212 | | GLY-TRP | 0.1078 | 0.0954 |
| GLU-TYR | 0.1298 | 0.1218 | | ASP-TRP | 0.1077 | 0.0973 |
| LEU-SER | 0.1298 | 0.1219 | | LEU-TRP | 0.1075 | 0.0964 |
| ALA-ARG | 0.1296 | 0.1232 | | TRP-VAL | 0.1071 | 0.0921 |
| PHE-SER | 0.1295 | 0.1187 | | MET-TYR | 0.1070 | 0.1062 |
| ARG-GLY | 0.1295 | 0.1227 | | ALA-TRP | 0.1067 | 0.0949 |
| LYS-TYR | 0.1295 | 0.1200 | | PHE-TRP | 0.1054 | 0.0962 |
| ASP-ILE | 0.1295 | 0.1182 | | MET-PHE | 0.1052 | 0.1091 |
| ASP-PRO | 0.1294 | 0.1243 | | HIS-TRP | 0.1050 | 0.0907 |
| ASP-LEU | 0.1293 | 0.1221 | | PRO-TRP | 0.1047 | 0.0948 |
| PRO-SER | 0.1293 | 0.1228 | | TRP-TYR | 0.1017 | 0.0898 |
| ARG-LEU | 0.1293 | 0.1227 | | CYS-GLU | 0.0988 | 0.0897 |
| ASP-VAL | 0.1291 | 0.1197 | | CYS-LYS | 0.0981 | 0.0875 |
| GLN-LEU | 0.1291 | 0.1229 | | ARG-CYS | 0.0972 | 0.0872 |
| LEU-THR | 0.1291 | 0.1214 | | MET-TRP | 0.0962 | 0.0875 |
| ILE-THR | 0.1287 | 0.1179 | | ASP-CYS | 0.0956 | 0.0861 |
| GLY-THR | 0.1286 | 0.1205 | | CYS-SER | 0.0954 | 0.0861 |
| ASN-VAL | 0.1283 | 0.1165 | | ASN-CYS | 0.0951 | 0.0861 |
| ARG-ILE | 0.1282 | 0.1201 | | CYS-GLN | 0.0945 | 0.0877 |
| ALA-ILE | 0.1282 | 0.1167 | | CYS-THR | 0.0936 | 0.0849 |
| LYS-PHE | 0.1282 | 0.1241 | | CYS-GLY | 0.0920 | 0.0845 |
| LYS-VAL | 0.1280 | 0.1202 | | CYS-PRO | 0.0920 | 0.0847 |
| ASN-PRO | 0.1279 | 0.1223 | | CYS-ILE | 0.0919 | 0.0830 |
| ALA-THR | 0.1276 | 0.1202 | | CYS-TYR | 0.0912 | 0.0832 |
| ILE-VAL | 0.1276 | 0.1153 | | CYS-PHE | 0.0911 | 0.0832 |
| GLN-ILE | 0.1275 | 0.1235 | | ALA-CYS | 0.0911 | 0.0859 |
| PHE-PRO | 0.1274 | 0.1180 | | CYS-LEU | 0.0910 | 0.0864 |
| SER-TYR | 0.1273 | 0.1185 | | CYS-VAL | 0.0907 | 0.0821 |
| ARG-PHE | 0.1273 | 0.1232 | | CYS-HIS | 0.0879 | 0.0797 |
| GLY-PRO | 0.1273 | 0.1211 | | CYS-MET | 0.0851 | 0.0818 |
| ASP-TYR | 0.1273 | 0.1182 | | CYS-TRP | 0.0805 | 0.0739 |
| ASN-HIS | 0.1272 | 0.1104 | | GLU-GLU | 0.0616 | 0.0593 |
| PHE-THR | 0.1271 | 0.1193 | | LYS-LYS | 0.0612 | 0.0565 |
| ARG-TYR | 0.1270 | 0.1193 | | SER-SER | 0.0606 | 0.0545 |
| ASN-ILE | 0.1268 | 0.1191 | | ASP-ASP | 0.0589 | 0.0543 |
| ASP-PHE | 0.1268 | 0.1203 | | ARG-ARG | 0.0587 | 0.0543 |
| HIS-SER | 0.1266 | 0.1143 | | LEU-LEU | 0.0578 | 0.0566 |
| GLN-HIS | 0.1265 | 0.1142 | | THR-THR | 0.0575 | 0.0518 |
| GLU-HIS | 0.1264 | 0.1182 | | ALA-ALA | 0.0561 | 0.0510 |
| GLN-TYR | 0.1263 | 0.1209 | | GLY-GLY | 0.0558 | 0.0528 |
| PRO-THR | 0.1263 | 0.1170 | | ASN-ASN | 0.0549 | 0.0507 |
| ILE-LEU | 0.1259 | 0.1207 | | GLN-GLN | 0.0543 | 0.0521 |
| GLN-PHE | 0.1258 | 0.1208 | | ILE-ILE | 0.0543 | 0.0516 |
| ARG-HIS | 0.1258 | 0.1144 | | VAL-VAL | 0.0542 | 0.0507 |
| ALA-PRO | 0.1257 | 0.1212 | | PRO-PRO | 0.0515 | 0.0483 |
| ASP-HIS | 0.1257 | 0.1135 | | PHE-PHE | 0.0502 | 0.0492 |
| ALA-VAL | 0.1255 | 0.1169 | | TYR-TYR | 0.0459 | 0.0437 |
| ALA-LEU | 0.1254 | 0.1174 | | HIS-HIS | 0.0415 | 0.0389 |
| ASN-TYR | 0.1252 | 0.1179 | | MET-MET | 0.0357 | 0.0367 |
| LEU-VAL | 0.1251 | 0.1199 | | TRP-TRP | 0.0317 | 0.0266 |
| HIS-LYS | 0.1251 | 0.1180 | | CYS-CYS | 0.0275 | 0.0241 |

**Supplementary Table S3.** Structural evaluation for proteins shown in Figure 5 and Figure 6 in the main text. For each protein, “pre” represents the structure from our predictions, “input1” and “input2” represent two input structures directly from Protein Data Bank (PDB). “input1_minimize” and “input2_minimize” represent the structures from PDB but subjected to an additional energy minimization step (please see “Minimization protocol” in the Method section) before evaluation. MolProbity score combines the clashscore, rotamer, and Ramachandran evaluations into a single indicator of model quality. The corresponding rank indicates the percentile relative to structures of similar resolution. MolProbity considers a rank higher than 66% as good.

| Protein | Molprobity score (percentile) | Poor rotamers | Ramachandran outliers | Favored rotamers | Ramachandran favored |
| --- | --- | --- | --- | --- | --- |
| RfaH_pre (state 2) | 87% | 2.38% | 0.00% | 90.48% | 86.96% |
| RfaH_pre (state 3) | 94% | 0.00% | 2.17% | 92.86% | 82.61% |
| RfaH_pre (state 4) | 73% | 0.00% | 0.00% | 97.62% | 82.61% |
| RfaH_input1 | 54% | 2.38% | 0.00% | 85.71% | 97.83% |
| RfaH_input2 | 30% | 2.38% | 2.17% | 88.10% | 95.65% |
| RfaH_input1_minimize | 99% | 2.38% | 0.00% | 97.62% | 97.83% |
| RfaH_input2_minimize | 98% | 2.38% | 0.00% | 88.10% | 89.13% |
|  |  |  |  |  |  |
| MurD_pre | 85.00% | 1.16% | 0.46% | 93.91% | 94.25% |
| MurD_input1 | 84.00% | 5.22% | 0.69% | 88.12% | 95.63% |
| MurD_input2 | 100.00% | 1.45% | 0.00% | 94.78% | 98.39% |
| MurD_input1_minimize | 84.00% | 0.58% | 0.92% | 96.23% | 93.33% |
| MurD_input2_minimize | 86.00% | 0.58% | 0.46% | 95.65% | 93.79% |
|  |  |  |  |  |  |
| KaiB_pre | 79% | 0.00% | 5.62% | 91.36% | 64.04% |
| KaiB_input1 | 79.00% | 6.17% | 3.37% | 85.19% | 87.64% |
| KaiB_input2 | 99.00% | 5.13% | 0.00% | 91.03% | 98.88% |
| KaiB_ input1_minimize | 77% | 1.23% | 1.12% | 97.53% | 94.38% |
| KaiB_ input2_minimize | 87% | 0.00% | 0.00% | 98.72% | 93.26% |
|  |  |  |  |  |  |
| myosin_pre | 87% | 3.51% | 1.64% | 90.64% | 86.51% |
| myosin_ input1 | 42.00% | 2.34% | 0.25% | 91.67% | 97.48% |
| myosin_ input2 | 100.00% | 1.02% | 0.25% | 96.93% | 97.10% |
| myosin_ input1_minimize | 78% | 1.46% | 0.88% | 95.03% | 92.18% |
| myosin_ input2_minimize | 89% | 1.02% | 1.26% | 97.08% | 92.06% |
|  |  |  |  |  |  |
| Protein | Clashscore | C-beta deviations > 0.25Å | Bad bond | Bad angle |  |
| RfaH_pre (state 2) | 1.27 | 0.00% | 0.00% | 0.57% |  |
| RfaH_pre (state 3) | 1.27 | 0.00% | 0.00% | 0.76% |  |
| RfaH_pre (state 4) | 6.35 | 0.00% | 0.00% | 0.76% |  |
| RfaH_ input1 | 38.12 | 0.00% | 0.26% | 1.53% |  |
| RfaH_ input2 | 59.72 | 0.00% | 0.00% | 1.15% |  |
| RfaH_ input1_minimize | 1.27 | 2.22% | 0.00% | 0.96% |  |
| RfaH_ input2_minimize | 0 | 0.00% | 0.00% | 0.57% |  |
|  |  |  |  |  |  |
| MurD_pre | 6.85 | 0.25% | 0.00% | 0.86% |  |
| MurD_ input1 | 2.13 | 0.50% | 0.06% | 0.73% |  |
| MurD_ input2 | 0.61 | 0.00% | 0.00% | 0.55% |  |
| MurD_ input1_minimize | 7.16 | 0.25% | 0.06% | 0.82% |  |
| Murd_ input2_minimize | 6.86 | 0.00% | 0.00% | 0.77% |  |
|  |  |  |  |  |  |
| KaiB_pre | 2.67 | 0.00% | 0.00% | 1.01% |  |
| KaiB_ input1 | 0.67 | 1.15% | 0.00% | 0.81% |  |
| KaiB_ input2 | 0.67 | 0.00% | 0.00% | 0.10% |  |
| KaiB_ input1_minimize | 10 | 0.00% | 0.00% | 0.30% |  |
| KaiB_ input2_minimize | 6.06 | 0.00% | 0.00% | 0.10% |  |
|  |  |  |  |  |  |
| myosin_pre | 0 | 0.94% | 0.18% | 1.11% |  |
| myosin_ input1 | 54.39 | 0.00% | 0.06% | 1.52% |  |
| myosin_ input2 | 0.16 | 0.00% | 0.06% | 0.11% |  |
| myosin_ input1_minimize | 6.29 | 0.13% | 0.05% | 0.96% |  |
| myosin_ input2_minimize | 4.17 | 0.13% | 0.05% | 0.83% |  |

**Supplementary Table S4.** Comparison of our model with several baseline architectures in predicting the distance matrix $D_{ij}$ for the transition state of protein conformational changes. 𝑟 represents the Pearson correlation coefficient between predictions and actual simulation data. MAE indicates the mean absolute error when the element of $D_{ij}$​ has no unit, as defined by eqn. (5) in the main text. MAE_dis represents the mean absolute error when the element of $D_{ij}$​ is the actual Euclidean distance. HESpre denotes our prediction. Structure A (Structure B) represents the case where the experimentally resolved structure A (structure B) is assumed as the guess for the transition state. SVM represents the machine learning method "Support Vector Machine". GNN represents the use of a permutation-equivariant graph neural network.

|  | **HESpre** | **Structure A** | **Structure B** | **SVM** | **GNN** |
| --- | --- | --- | --- | --- | --- |
| ***r*** | **0.77** | 0.44 | 0.39 | 0.60 | 0.67 |
| **MAE** | **0.044** | 0.062 | 0.080 | 0.098 | 0.063 |
| **MAE_dis (nm)** | **0.224** | 0.324 | 0.414 | 0.449 | 0.303 |
